# Synonymous and Nonsynonymous Substitutions in Dictyostelium discoideum Ammonium Transporter *amtA* Are Necessary for Functional Complementation in Saccharomyces cerevisiae

**DOI:** 10.1128/spectrum.03847-22

**Published:** 2023-02-22

**Authors:** Asha Densi, Revathi S. Iyer, Paike Jayadeva Bhat

**Affiliations:** a Department of Biosciences and Bioengineering, Indian Institute of Technology Bombay, Mumbai, India; University at Buffalo, State University of New York

**Keywords:** horizontal gene transfer, protein evolution, gene duplication, membrane proteins, single nucleotide polymorphism

## Abstract

Ammonium transporters are present in all three domains of life. They have undergone extensive horizontal gene transfer (HGT), gene duplication, and functional diversification and therefore offer an excellent paradigm to study protein evolution. We attempted to complement a *mep1*Δ*mep2*Δ*mep3*Δ strain of Saccharomyces cerevisiae (triple-deletion strain), which otherwise cannot grow on ammonium as a sole nitrogen source at concentrations of <3 mM, with *amtA* of Dictyostelium discoideum, an orthologue of S. cerevisiae *MEP2*. We observed that *amtA* did not complement the triple-deletion strain of S. cerevisiae for growth on low-ammonium medium. We isolated two mutant derivatives of *amtA* (*amtA M1* and *amtA M2*) from a PCR-generated mutant plasmid library that complemented the triple-deletion strain of S. cerevisiae. *amtA M1* bears three nonsynonymous and two synonymous substitutions, which are necessary for its functionality. *amtA M2* bears two nonsynonymous substitutions and one synonymous substitution, all of which are necessary for functionality. Interestingly, AmtA M1 transports ammonium but does not confer methylamine toxicity, while AmtA M2 transports ammonium and confers methylamine toxicity, demonstrating functional diversification. Preliminary biochemical analyses indicated that the mutants differ in their conformations as well as their mechanisms of ammonium transport. These intriguing results clearly point out that protein evolution cannot be fathomed by studying nonsynonymous and synonymous substitutions in isolation. The above-described observations have significant implications for various facets of biological processes and are discussed in detail.

**IMPORTANCE** Functional diversification following gene duplication is one of the major driving forces of protein evolution. While the role of nonsynonymous substitutions in the functional diversification of proteins is well recognized, knowledge of the role of synonymous substitutions in protein evolution is in its infancy. Using functional complementation, we isolated two functional alleles of the D. discoideum ammonium transporter gene (*amtA*), which otherwise does not function in S. cerevisiae as an ammonium transporters. One of them is an ammonium transporter, while the other is an ammonium transporter that also confers methylammonium (ammonium analogue) toxicity, suggesting functional diversification. Surprisingly, both alleles require a combination of synonymous and nonsynonymous substitutions for their functionality. These results bring out a hitherto-unknown pathway of protein evolution and pave the way for not only understanding protein evolution but also interpreting single nucleotide polymorphisms (SNPs).

## INTRODUCTION

Ammonium transporters belong to the Amt/Mep/Rh superfamily, members of which are distributed in all domains of life (1). Members of this family have undergone extensive duplication, sub- and neofunctionalization, gene fusion, and horizontal gene transfer (HGT) across kingdoms (2). For example, it has been proposed that a few species of archaea acquired Rh genes through HGT from eucaryotes (3). An independent bioinformatics analysis indicated that members of the Amt family are vertically transmitted, while members of the Mep family have undergone extensive HGT (4, 5). In general, ammonium transporters from any one of the families are represented in most taxa, except amoebae and nematodes, which harbor members belonging to both the Amt and Rh families (6). Members of the Mep family are present only in fungi, while vascular plants have only members of the Amt family (5). Members of the Amt/Mep/Rh superfamily are known to transport methylammonium and CO_2_ (3, 7–11) and also function as signal transducers in response to ammonium depletion (12). The amino acid sequence identity among members of this superfamily is less than 25% (13), thus making it difficult to track the evolutionary trajectory of functional diversification.

Saccharomyces cerevisiae has been the forerunner in studying the structure-function relationships (14, 15) and tracking the evolutionary trajectory (16, 17) of ammonium transporters. S. cerevisiae has three ammonium transporters, *MEP1*, *MEP2*, and *MEP3*. Only when all three transporters are deleted (triple-deletion strain) is S. cerevisiae unable to grow in a medium containing ammonium as a sole nitrogen source at concentrations of <3 mM (here, ammonium refers to the sum of NH_4_^+^ and NH_3_ unless otherwise mentioned). While functional diversification seems to have occurred among these three ammonium transporters, its biological significance is unclear. For example, only *MEP2* is a transceptor (12), meaning that it transports ammonium and mediates pseudohyphal differentiation, and it has the highest affinity for ammonium (8). An S. cerevisiae strain bearing *MEP1* alone exhibits toxicity in the presence of methylamine (7, 8, 15), suggesting that *MEP2* and *MEP3* are unable to transport methylamine. While there is considerable controversy regarding the mechanism of ammonium transport, preliminary reports suggest that the mechanism of transport is electrogenic and electroneutral in Mep1 and Mep2, respectively (18).

Studies conducted using experimental evolution of *Saccharomyces* in a limited-nitrogen environment have yielded intriguing results. It was observed that when interspecific diploids formed between S. cerevisiae and Saccharomyces uvarum that were evolved in a nitrogen-limited environment, interspecific rearrangements occurred several times in independent lines only within the *MEP2* locus (16). Experimental evolution of haploid S. cerevisiae in a limited-nitrogen environment resulted in the evolution of gene network polymorphisms, of which *MEP2* is one of the members (17). Thus, it is not surprising that with nitrogen being the second most important nutrient after carbon, ammonium transporters could have undergone extensive “molecular tinkering” (19) to face environmental constraints during their evolutionary sojourns.

The virulence of plant and animal pathogens of fungal origin has often been attributed to their ability to switch their developmental pathways in response to nitrogen deprivation (20). Ammonium transport coupled with cellular differentiation is widespread in evolutionarily diverse organisms, including plants (21, 22) and amoebae (23). We reasoned that studying the ammonium transporters of Dictyostelium discoideum, which diverged 1,480 million years ago from the metazoan lineage after plants but before yeasts (24), could provide insights into the functional diversification of ammonium transporters. The reasoning was that while these two organisms share some features such as cellularity (25, 26), they exhibit many distinct differences. For example, D. discoideum is AT rich, while S. cerevisiae is not. The D. discoideum genome carries the genes for five different putative ammonium transporters, *amtA*, *amtB*, *amtC*, *rhgA*, and *rhgB*. Of these, *amtA* and *amtC* have been studied with respect to differentiation. An *amtA*-deleted strain has an increased level of intracellular ammonium and a decreased level of extracellular ammonium. This suggests that AmtA is involved in exporting ammonium out of the cell (23, 27). Despite this difference, an *amtA* deletion mutant of D. discoideum is defective in cellular differentiation (23), suggesting a common evolutionary pathway of cellular differentiation between these two organisms in response to ammonium depletion. If so, the question is, How is function conserved in the face of such evolutionary divergence?

Previous studies have used complementation approaches to study the structure-function relationships of ammonium transporters from humans (28), plants (29), Caenorhabditis elegans (30), and Escherichia coli (14). These proteins readily complemented the triple-deletion strain for growth on low-ammonium medium. We surmised that a complementation strategy like the one described above can help us understand the structure-function relationship of the ammonium transporter *amtA* from D. discoideum. Surprisingly, unlike previous reports (14, 28–30), *amtA* of D. discoideum failed to complement the triple-deletion strain for growth on ammonium at concentrations of <3 mM as the sole source of nitrogen. This defect could not be ascribed to possibilities such as impaired synthesis or membrane localization. This forced us to isolate mutants of *amtA* that complemented the triple-deletion strain for growth on low-ammonium medium. We observed that in these mutants, a combination of synonymous and nonsynonymous mutations is necessary to confer ammonium uptake activity to AmtA in S. cerevisiae. These observations challenge the long-held notion that “second-site functional compensation” (which occurs when a loss-of-function mutation is suppressed by a second-site mutation) in a protein occurs only through nonsynonymous substitutions. The implications of these observations for protein evolution are discussed in detail.

## RESULTS

### D. discoideum *amtA* does not complement the S. cerevisiae triple-deletion strain for growth on minimal medium containing ammonium at concentrations below 3 mM.

**FIG 1:**
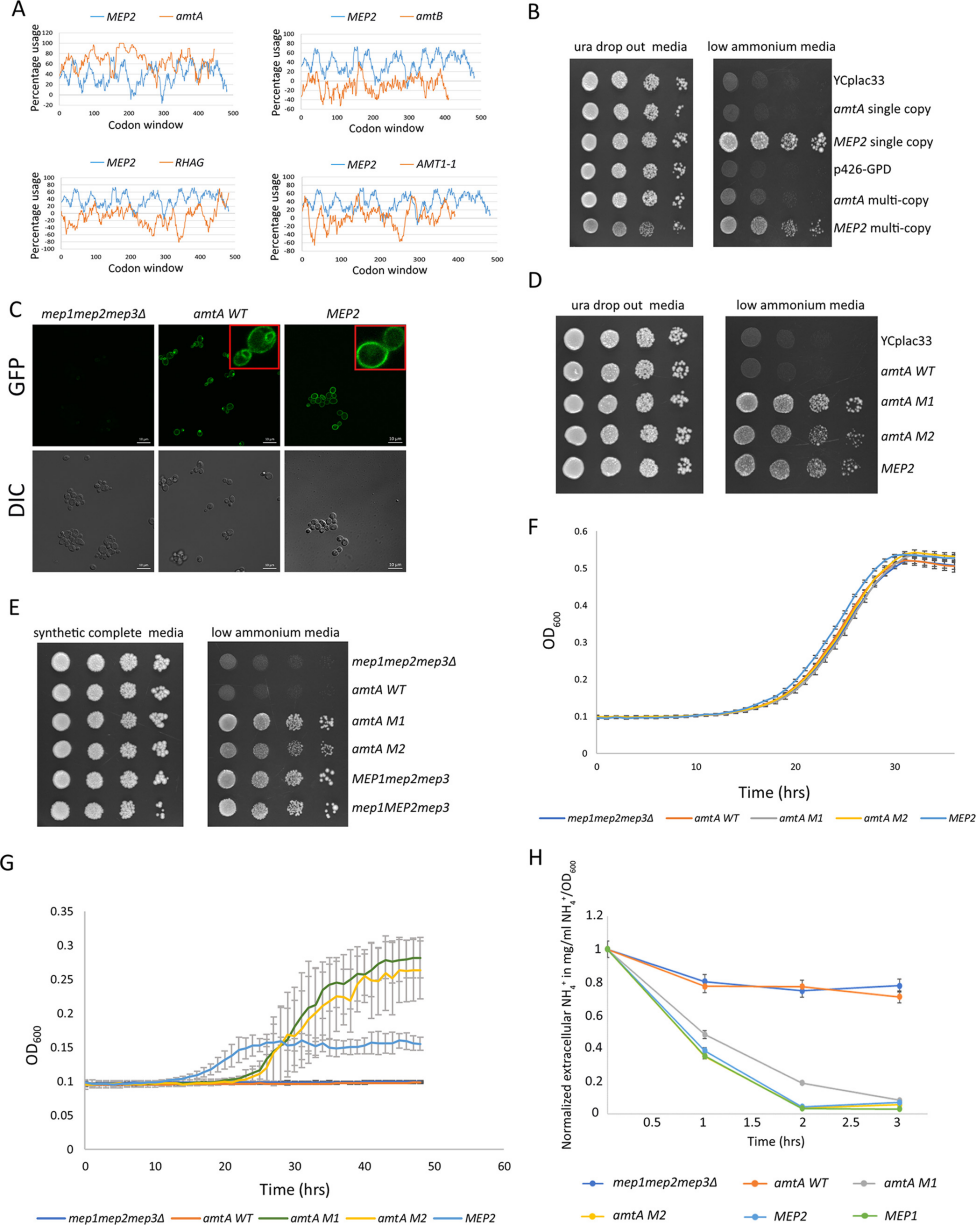
*amtA M1* and *amtA M2*, but not *amtA WT*, complement a triple-deletion strain. (A) %MinMax of ammonium transporters expressed in S. cerevisiae. %MinMax values of ammonium transporters expressed in S. cerevisiae were obtained using %MinMax calculator online software (32, 33). The percent usage of rare codons was plotted against a sliding codon window of 18 codons. (B) Growth of transformants on low-ammonium medium. Five microliters of transformants of the triple-deletion strain pregrown in Ura dropout glucose medium containing 20 mM ammonium to a cell density of 10^7^ cells/mL was serially diluted and spotted onto Ura dropout glucose medium with 20 mM ammonium sulfate (left) and low-ammonium medium (right). The growth pattern was photographed after 4 days of incubation at 30°C. (C) Expression and localization of yEGFP-tagged proteins. Strains with yEGFP-tagged proteins were grown in 0.1% proline medium for 22 h. The localization of the yEGFP-tagged proteins was visualized using live fluorescence microscopy. (Top) GFP fluorescence images; (bottom) differential interference contrast (DIC) images. (D) Growth of transformants on low-ammonium medium. Five microliters of transformants of the triple-deletion strain pregrown in Ura dropout glucose medium containing 20 mM ammonium to a cell density of 10^7^ cells/mL was serially diluted and spotted onto Ura dropout glucose medium with 20 mM ammonium sulfate (left) and low-ammonium medium (right). The growth pattern was photographed after 4 days of incubation at 30°C. (E) Growth of strains on low-ammonium medium. Five microliters of strains pregrown in synthetic complete glucose medium containing 20 mM ammonium to a cell density of 10^7^cells/mL was serially diluted and spotted onto synthetic complete glucose medium with 20 mM ammonium sulfate (left) and low-ammonium medium (right). The growth pattern was photographed after 4 days of incubation at 30°C. (F) Growth kinetics of strains on 0.1% proline medium. Strains pregrown in 0.1% proline medium were grown in 0.1% proline medium, and the OD_600_ was measured as a function of time for 36 h. (G) Growth kinetics of strains on low-ammonium medium. Strains pregrown in 0.1% proline medium were grown in low-ammonium medium, and the OD_600_ was measured as a function of time for 48 h. (H) External ammonium concentration (in milligrams per milliliter of NH_4_^+^/OD_600_) as a function of time. Different strains were grown in 0.1% proline medium until the OD_600_ reached 1.0. Cells were collected and transferred to a medium containing 0.1% proline and 500 μM ammonium at an OD_600_ of 1.0. At each time point, cells were collected and filtered, and the ammonium concentration in the filtrate was estimated as a function of time.

Functional complementation is normally used as a proxy to investigate the genetic relatedness between species and decipher the structure-function relationships of proteins (31). For example, ammonium transporters from humans (28), plants (29), worms (30), and bacteria (14) functionally complement an S. cerevisiae triple-deletion strain that otherwise does not grow on synthetic medium containing ammonium at concentrations of <3 mM. Ammonium transporters of both D. discoideum and S. cerevisiae play a role in cellular differentiation. However, their genomes are significantly different in GC content (22.4% in D. discoideum versus 38% in S. cerevisiae). Therefore, it was imperative to look at the codon adaptive index (CAI) and the relative usage frequencies of synonymous codons (%MinMax) of the ammonium transporters of these two organisms and compare these values to those of the ammonium transporters of organisms whose ammonium transporters are known to complement the S. cerevisiae triple-deletion strain (32, 33). The CAIs of *RHAG* (Homo sapiens), *AMT1-1* (Arabidopsis thaliana), and *amtB* (E. coli) were lower than those of *MEP2* in the context of the S. cerevisiae genome (Table 1). Similarly, the %MinMax values were lower for *RHAG* (H. sapiens versus S. cerevisiae), *AMT1-1* (A. thaliana versus S. cerevisiae), and *amtB* (E. coli versus S. cerevisiae) (Fig. 1A). Surprisingly, both the CAI and %MinMax values of *amtA* (D. discoideum versus S. cerevisiae) were higher than those of *MEP2*. The above-described analysis suggested that D. discoideum *amtA* codons are more favorable for translation in S. cerevisiae than *RHAG*, *AMT1-1*, and *amtB*. Based on these bioinformatics analyses, we hypothesized that the difference in GC content observed between D. discoideum and S. cerevisiae should not impair AmtA functionality in S. cerevisiae.

**TABLE 1:** CAIs of different ammonium transporters expressed in S. cerevisiae

Ammonium transporter	CAI
S. cerevisiae *MEP2*	0.78
D. discoideum *amtA*	0.86
E. coli *amtB*	0.58
*A. thaliana AMT1-1*	0.62
H. sapiens *RHAG*	0.67

The triple-deletion strain was transformed separately with a single-copy (YCplac33) or a multicopy (p426-GPD) plasmid bearing either *amtA* or *MEP2*, whose expression was driven by the *MEP2* promoter. These transformants were pregrown in uracil (Ura) dropout medium containing ammonium sulfate at a concentration of 20 mM to a cell density of 5 × 10^7^ cells/mL, serially diluted, and spotted onto uracil dropout medium and minimal medium containing ammonium at a concentration of 500 μM as the sole nitrogen source (referred to as low-ammonium medium here) (Fig. 1B). The transformant bearing the YCplac33 vector showed no growth, while the positive control bearing either single-copy or multicopy *MEP2* showed growth as expected. It was observed that the triple-deletion strain transformed with multicopy *MEP2* consistently showed slight growth retardation compared to the growth of the transformant bearing single-copy *MEP2*, even on complete medium (Fig. 1B). Transformants of the triple-deletion strain bearing single-copy *amtA* or multicopy *amtA* did not grow on low-ammonium medium. No colonies were observed when the triple-deletion strain transformed with either single-copy or multicopy *amtA* was streaked for single colonies (see Fig. S1 in the supplemental material), clearly indicating that AmtA does not function as an ammonium transporter in S. cerevisiae.

The above-described results were unexpected as orthologues of ammonium transporters from other evolutionarily distant species complement the triple-deletion strain for growth on low-ammonium medium (14, 28–30). Furthermore, despite the codon usage of D. discoideum being favorable, *amtA* failed to complement the triple-deletion strain. We wanted to ascertain whether the incompatibility observed in our experiments is due to a defect in expression and/or plasma membrane localization. To test this, a strain with the yeast enhanced green fluorescent protein (GFP) gene (*yEGFP*) fused to the C terminus of *amtA* integrated at the *MEP2* locus was constructed such that the expression of *amtA*::*GFP* was driven by the *MEP2* promoter. *yEGFP* was similarly integrated into the C terminus of *MEP2*. These strains were grown for 22 h in 0.1% proline, a poor nitrogen source. Under these growth conditions, the *MEP2* promoter is transcriptionally active (34). Live cells were observed under a confocal microscope (Fig. 1C). A GFP signal was detected in the plasma membrane of *amtA*::*GFP* strain cells. In addition, a GFP signal was also detected in the subcellular compartment in the cells. As expected, it was observed that the GFP signal was localized primarily in the plasma membrane in the strain expressing the *MEP2*::*GFP* construct. Based on this, we inferred that the inability of *amtA* to complement a triple-deletion yeast strain on low-ammonium medium may not be because of a defect in expression or localization.

Based on the above-described results, we hypothesized that, most likely, the conformation of AmtA when expressed in S. cerevisiae is incompatible with its normal function. This could be because of aberrant cotranslational folding leading to a nonfunctional protein (35) or could be due to differences in the lipid composition of the membrane, which can influence the conformation of proteins (36). Using substitutions with frequently used codons to increase the translation rate, reducing GC content to reduce the possibility of stable mRNA secondary structures, or even generating a harmonized gene variant are some of the commonly used strategies to improve the expression/functionality of heterologously expressed genes (37–40). We reasoned that the above-described brute-force approaches for obtaining a functional protein would not give us insights into the trajectory of protein evolution. Therefore, we resorted to a reverse genetic approach. We constructed two independent mutant libraries of *amtA* in a single-copy plasmid using two independent error-prone PCRs (EP-PCRs) (see Materials and Methods for details), followed by ligation through *in vivo* recombination (Fig. S2). The expression of mutant open reading frames (ORFs) was driven by the *MEP2* promoter. Transformants generated as a result of *in vivo* recombination were selected on uracil dropout medium containing 20 mM ammonium (41). These transformants were replica plated onto low-ammonium medium. Approximately 25,000 transformants generated from a PCR library using 0.35 mM MnCl_2_ and 0.45 mM MnCl_2_ each were screened (total of 50,000 transformants). We obtained three transformants that grew on low-ammonium medium, of which one transformant was from the library generated using 0.35 mM MnCl_2_ and two were from the library generated using 0.45 mM MnCl_2_. However, we could recover only one transformant from each library. The mutant plasmid clone recovered from the library generated using 0.35 mM MnCl_2_ is referred to as *amtA M1*, and the mutant plasmid clone recovered from the library generated using 0.45 mM MnCl_2_ is referred to as *amtA M2*. Plasmids isolated from these transformants were amplified in E. coli, and the triple-deletion strain was retransformed with *amtA M1* and *amtA M2* independently. The growth phenotypes of these two transformants were observed using spot assays (Fig. 1D) and by streaking for single colonies on low-ammonium medium (Fig. S3). Thus, it was confirmed that the triple-deletion strain transformed with these mutants grew on low-ammonium medium. Wild-type *amtA* (referred to as *amtA WT* here), *amtA M1*, and *amtA M2* were integrated at the *MEP2* locus such that expression was driven by the *MEP2* promoter. The ability of these strains to grow on low-ammonium medium was tested by spot assays (Fig. 1E). These results are identical to those obtained using single-copy plasmid transformants.

We then carried out growth kinetics analyses of these strains grown in minimal medium containing 0.1% proline as the sole nitrogen source and on low-ammonium medium (Fig. 1F and G). Growth in minimal medium containing 0.1% proline as the sole nitrogen source was monitored at intervals of 1 h for a period of 36 h (Fig. 1F). All strains showed similar growth patterns, with a growth rate of 0.11 h^−1^. For the growth assay in low-ammonium medium (Fig. 1G), the experiment was carried out for 48 h. The triple-deletion strain and the strain expressing *amtA WT* did not show any growth. The strain expressing *MEP2* grew at a growth rate of 0.03 h^−1^. In comparison, strains expressing *amtA M1* and *amtA M2* grew at growth rates of 0.1 h^−1^ and 0.08 h^−1^, respectively. The strain expressing *MEP2* showed a growth lag of approximately 12 h, whereas the strains expressing *amtA M1* and *amtA M2* showed a growth lag of approximately 20 h. Furthermore, the final biomass of the strain expressing *MEP2* was much lower than those of the strains expressing *amtA M1* and *amtA M2* at saturation. The observed difference in the growth profiles of the *MEP2* and *amtA* mutants clearly reflects a difference in their functionality as ammonium transporters. However, the underlying cause of the observed difference is difficult to decipher without further experimentation.

We also determined the ability of strains expressing *amtA WT*, *amtA M1*, *amtA M2*, and *MEP2* to take up ammonium from the medium as a function of time (Fig. 1H). Strains were grown in low-ammonium medium, and the ammonium remaining in the medium as a function of time was estimated. While the triple-deletion strain or the strain expressing *amtA WT* failed to take up ammonium (Fig. 1H), the *amtA M1* and *amtA M2* strains showed ammonium uptake activity comparable to that of the *MEP2* strain. At the end of 3 h, the ammonium concentrations were close to zero in the media in which the strains expressing *amtA M1*, *amtA M2*, *MEP1*, and *MEP2* were grown. The external ammonium concentration remained close to the initial concentration in the media in which the triple-deletion strain and the strain expressing *amtA WT* were grown. This observation is in concordance with the growth phenotype on low-ammonium medium where only strains expressing *amtA M1*, *amtA M2*, *MEP1*, and *MEP2* grew.

### Both synonymous and nonsynonymous substitutions are necessary to confer functionality to *amtA M1* and *amtA M2*.

**FIG 2:**
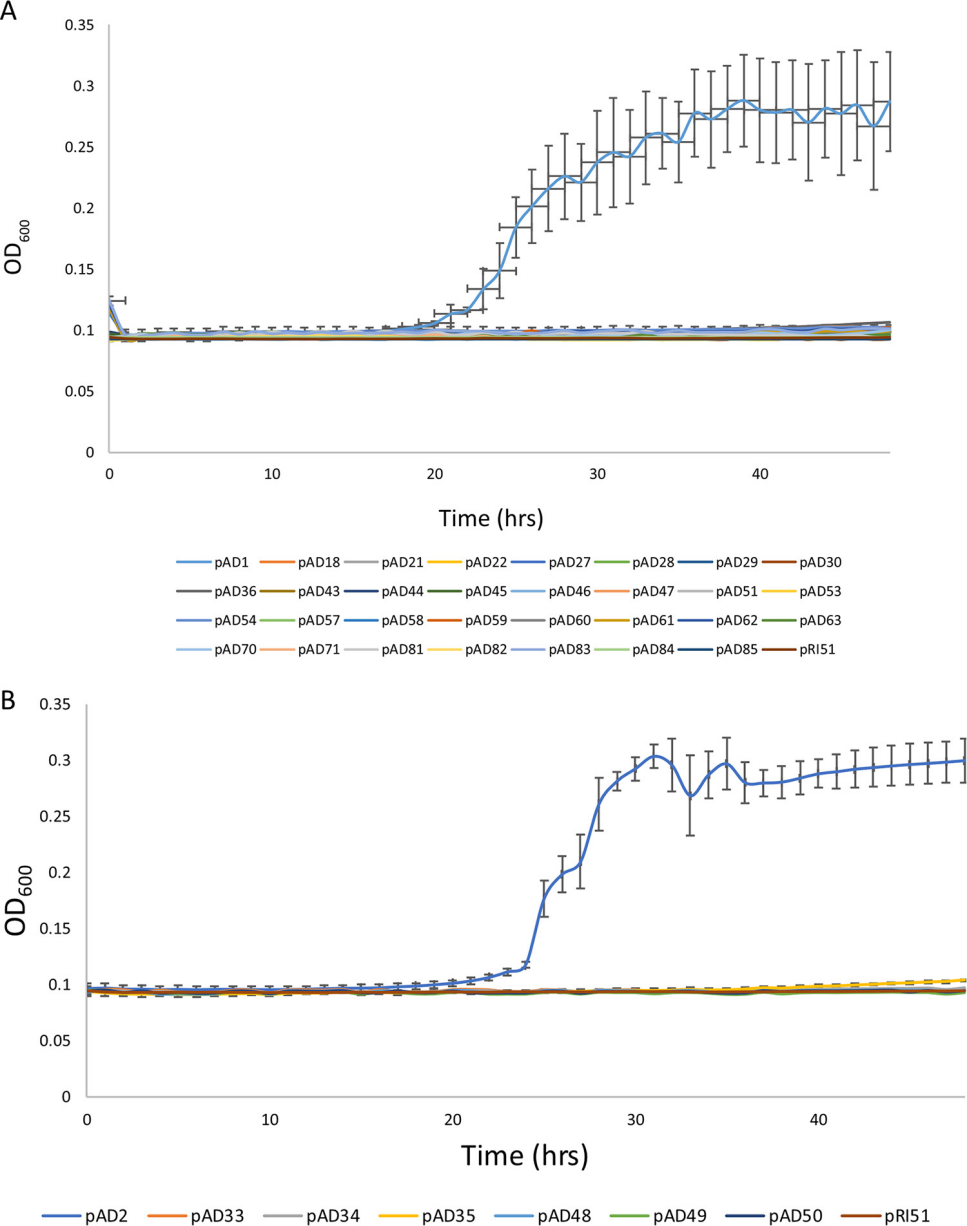
A combination of synonymous and nonsynonymous mutations is essential for *amtA M1* and *amtA M2* functionality. (A) Growth kinetics of transformants on low-ammonium medium. Transformants pregrown in 0.1% proline medium were grown in low-ammonium medium, and the OD_600_ was measured as a function of time for 48 h. Each line represents a transformant bearing a plasmid clone with a specific substitution mutation(s) in *amtA WT* corresponding to *amtA M1*. The resulting substitutions are as follows: pAD1, *amtA_Q89L_*_,_*_A134V_*_,_*_I301I_*_,_*_S311T_*_,_*_S350S_*; pAD18, *amtA_Q89L_*; pAD21, *amtA_A134V_*; pAD22, *amtA_S311T_*; pAD27, *amtA_Q89L_*_,_*_S350S_*; pAD28, *amtA_Q89L_*_,_*_A134V_*; pAD29, *amtA_A134V_*_,_*_S311T_*; pAD30, *amtA_Q89L_*_,_*_S311T_*; pAD36, *amtA_Q89L_*_,_*_A134V_*_,_*_S311T_*; pAD43, *amtA_A134V_*_,_*_I301I_*; pAD44, *amtA_I301I_*; pAD45, *amtA_Q89L_*_,_*_A134V_*_,_*_I301I_*_,_*_S311T_*; pAD46, *amtA_S350S_*; pAD47, *amtA_Q89L_*_,_*_A134V_*_,_*_S311T_*_,_*_S350S_*; pAD51, *amtA_I301I_*_,_*_S350S_*; pAD53, *amtA_A134V_*_,_*_S350S_*; pAD54, *amtA_Q89L_*_,_*_A134V_*_,_*_I301I_*_,_*_S311T_*_,_*_S350S_*; pAD57, *amtA_S311T_*_,_*_S350S_*; pAD58, *amtA_A134V_*_,_*_I301I_*_,_*_S311T_*; pAD59, *amtA_I301I_*_,_*_S311T_*; pAD60, *amtA_I301I_*_,_*_S311T_*_,_*_S350S_*; pAD61, *amtA_Q89L_*_,_*_I301I_*_,_*_S350S_*; pAD62, *amtA_A134V_*_,_*_I301I_*_,_*_S350S_*; pAD63, *amtA_A134V_*_,_*_S311T_*_,_*_S350S_*; pAD70, *amtA_Q89L_*_,_*_A134V_*_,_*_S350S_*; pAD71, *amtA_Q89L_*_,_*_A134V_*_,_*_I301I_*; pAD81, *amtA_Q89L_*_,_*_I301I_*_,_*_S311T_*; pAD82, *amtA_Q89L_*_,_*_S311T_*_,_*_S350S_*; pAD83, *amtA_A134V_*_,_*_I301I_*_,_*_S311T_*_,_*_S350S_*; pAD84, *amtA_Q89L_*_,_*_I301I_*_,_*_S311T_*_,_*_S350S_*; pAD85, *amtA_Q89L_*_,_*_A134V_*_,_*_I301I_*_,_*_S350S_*; and pRI51, *amtA WT*. (B) Growth kinetics of transformants on low-ammonium medium. Different transformants pregrown in 0.1% proline medium were grown in low-ammonium medium, and the OD_600_ was measured as a function of time for 48 h. Each line represents a transformant bearing a plasmid clone with a specific substitution mutation(s) in *amtA WT* corresponding to *amtA M2*. The resulting substitutions are as follows: pAD2, *amtA_P129T_*_,_*_K288K_*_,_*_V334I_*; pAD33, *amtA_P129T_*; pAD34, *amtA_V334I_*; pAD35, *amtA_P129T_*_,_*_V334I_*; pAD48, *amtA_K288K_*; pAD49, *amtA_P129T_*_,_*_K288K_*; pAD50, *amtA_K288K_*_,_*_V334I_*; and pRI51, *amtA WT*.

A comparison of the nucleotide sequences of *amtA WT*, *amtA M1*, and *amtA M2* indicated that *amtA M1* has three nonsynonymous (Q89L, A134V, and S311T) and two synonymous (I301I and S350S) mutations and that *amtA M2* has two nonsynonymous mutations (P129T and V334I) and one synonymous mutation (K288K). Thirty-one and seven different combinations of these mutations are possible in *amtA M1* and *amtA M2*, respectively. By site-directed mutagenesis, plasmid clones bearing these combinations of nonsynonymous and synonymous substitutions of *amtA M1* and *amtA M2*, respectively, were generated. The functionality of these mutant clones was tested by carrying out a growth kinetics assay by growing the transformants on low-ammonium medium, as described above (Fig. 2). Plasmid clones of *amtA M1* bearing individual nonsynonymous or synonymous substitutions did not grow on low-ammonium medium (Fig. 2A). Nonsynonymous substitutions in groups of either two or three also did not grow. Similarly, synonymous mutations either alone or together did not confer activity. Two nonsynonymous mutations and one synonymous mutation in all combinations did not confer functionality. Three nonsynonymous substitutions with any one of the synonymous substitutions also did not show activity. Functionality was observed only when all three nonsynonymous and two synonymous substitutions were present together (Fig. 2A). The above-described analysis indicates that both synonymous and nonsynonymous substitutions are necessary to confer functionality to *amtA M1*.

A similar analysis was carried out with *amtA M2*. It was observed that plasmid clones bearing either nonsynonymous or synonymous mutations of *amtA M2* individually did not grow on low-ammonium medium (Fig. 2B). The combination of any of the two mutations, synonymous or nonsynonymous, did not confer functionality. Functionality was observed only when the two nonsynonymous mutations and the one synonymous mutation were all present (Fig. 2B). These observations clearly demonstrate that nonsynonymous or synonymous mutations in isolation do not confer functionality and that a combination of both is necessary for the function of *amtA M1* as well as *amtA M2*. To the best of our knowledge, this is the first report where a gain of function is dependent on a combination of both types of substitutions.

### Wild-type AmtA and its mutant derivatives exhibit differential behavior in the plasma membrane and sensitivity to proteolytic cleavage.

**FIG 3:**
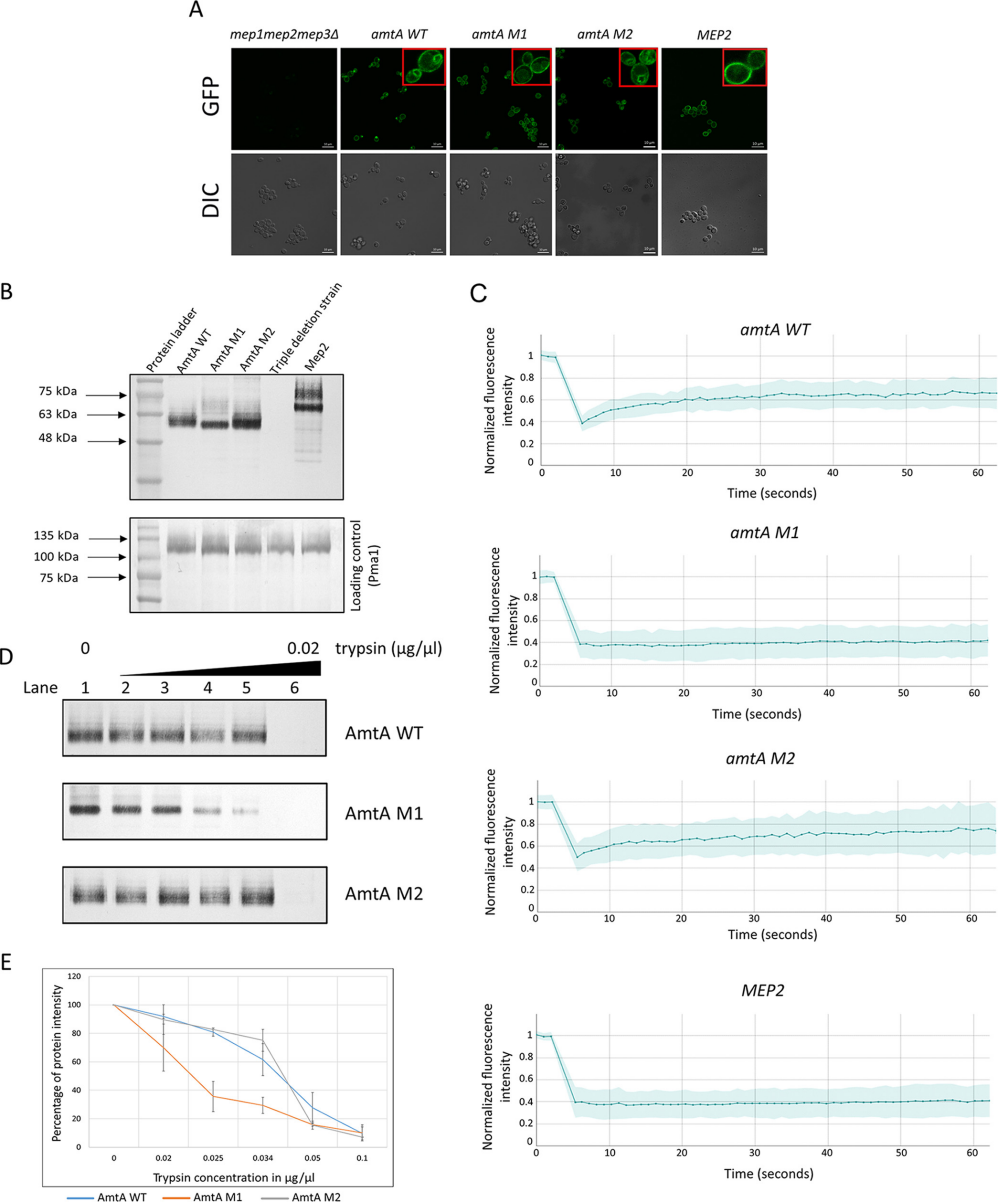
*amtA WT*, *amtA M1*, and *amtA M2* differ in their proteolytic sensitivities and plasma membrane mobilities. (A) Expression and localization of yEGFP-tagged proteins. Strains with yEGFP-tagged proteins were grown in 0.1% proline medium for 22 h. The localization of the yEGFP-tagged proteins was visualized using live fluorescence microscopy. (Top) GFP fluorescence images; (bottom) DIC images. (B) Protein expression in the membrane fraction. The membrane fraction was isolated from strains grown in 0.1% proline medium for 22 h, and Western blotting was performed. Anti-GFP antibody was used to detect the GFP-tagged protein. Pma1 antibody was used to detect the Pma1 protein that served as a loading control. (C) Fluorescence recovery after photobleaching (FRAP). yEGFP-tagged *amtA WT*, *amtA M1*, *amtA M2*, and *MEP2* strains were subjected to FRAP for 60 s. The fluorescence intensity at each data point is normalized to the fluorescence intensity at photobleaching. (D) Digestion pattern after trypsin digestion of the membrane fraction. Membrane fractions of the *amtA WT*, *amtA M1*, *amtA M2*, and *MEP2* strains were subjected to trypsin digestion. No trypsin was used in lane 1, and increasing concentrations of trypsin, 0.004 μg/μL, 0.005 μg/μL, 0.0067 μg/μL, 0.01 μg/μL, and 0.02 μg/μL, were used from the 2nd lane onward. (E) Quantitative analysis of protease digestion patterns after trypsin digestion of membrane fractions. Membrane fractions of the *amtA WT*, *amtA M1*, *amtA M2*, and *MEP2* strains were subjected to trypsin digestion. The percent protein intensity with respect to the protein intensity present without trypsin is plotted against the trypsin concentration.

The membrane localization patterns of AmtA M1 and AmtA M2 were studied by using strains with yEGFP tagged at the C termini of the respective proteins. The functionality of the mutants after yEGFP tagging was confirmed by testing their ability to grow on low-ammonium medium (Fig. S4). Tagging yEGFP at the C terminus of AmtA mutant derivatives did not hamper their biological function (Fig. S4). GFP fluorescence signals were detected in the plasma membranes of the *amtA M1*::*GFP* and *amtA M2*::*GFP* strains (Fig. 3A). A GFP signal was also observed in the subcellular compartment of the strain expressing *amtA M2*::*GFP*, similar to what was observed for the strain expressing *amtA WT*::*GFP* (Fig. 3A). The subcellular localization of the human ammonium transporters RhAG and RhCG in S. cerevisiae was reported previously (42). It is not clear whether the subcellular GFP signal observed in cells expressing *amtA WT* and *amtA M2* is a degraded product or the fusion protein.

To corroborate the observations that AmtA and its derivatives are localized in the plasma membrane, we carried out subcellular isolation of the plasma membrane from strains grown in 0.1% proline, followed by Western blotting using antibodies against GFP (Fig. 3B). It is pertinent to mention that the membrane fraction was treated with SDS and 8 M urea and incubated at room temperature instead of being heated at 100°C before it was subjected to SDS electrophoresis, to avoid protein aggregation (43). The plasma membrane ATPase 1 (Pma1) protein was used as a loading control. It can be observed that AmtA WT, AmtA M1, and AmtA M2 are present in the membrane fraction (Fig. 3B). The normalized intensities of AmtA WT, AmtA M1, and AmtA M2 with respect to Pma1 were found to be 1.5, 1.6, and 2.1, respectively. The expected molecular weight of the AmtA fusion protein is 77 kDa. The observed molecular weight obtained from the above-described data is 62 kDa. It is commonly observed that membrane proteins exhibit anomalous mobilities on SDS-PAGE gels to an extent of approximately 30% (44).

It was previously reported that synonymous codons can result in the synthesis of conformationally heterogeneous protein products (45). This can result in differences in disposition between AmtA M1 and AmtA M2. For example, ammonium transporters are known to exist as trimers, and a difference in the subunit conformation can affect trimerization (46, 47). It is also possible that the lipid-protein interaction in the plasma membrane could have a deleterious effect on functionality because of a difference in the dispositions of AmtA M1 and AmtA M2 in the plasma membrane. We decided to probe the above-mentioned possibilities by carrying out experimental analyses to determine if there are any differences in the conformations of the proteins and, by extension, differences in the lateral diffusion of the proteins in the plasma membrane. Fluorescence recovery after photobleaching (FRAP) analysis was performed to determine whether there is a difference in lateral diffusion among AmtA WT, AmtA M1, and AmtA M2 in the plasma membrane, in milieu in which they are functional. The pattern of recovery after photobleaching was determined by measuring the normalized fluorescence intensity as a function of time. The measurement was done for a total of 60 s on 37 samples for each strain (Fig. 3C). We observed that AmtA M1 and Mep2 failed to recover after photobleaching, with recovery rates of 4.76% and 6.54%, respectively, suggesting that their mobility was restricted. On the other hand, AmtA WT recovered to the extent of 41.77%, while AmtA M2 recovered to the extent of 48.82%. This suggests that both AmtA WT and AmtA M2 have a mobile fraction. The above-described results suggest that functionality does not necessarily reflect the recovery rate.

To further probe the differences among AmtA WT, AmtA M1, and AmtA M2, we looked at the sensitivity of these proteins to trypsin digestion. If there are conformational differences between AmtA WT and the mutants, the kinetics of digestion in the presence of various concentrations of trypsin are expected to vary. The kinetics were monitored by probing the proteins using GFP antibody by Western blotting, as GFP does not get digested by trypsin (48, 49). Thus, the difference in the kinetics of trypsin digestion, if any, ought to be due to a difference in the conformations of the proteins. Figure 3D shows a representative result from the experiment. Data from an average of four experiments are presented in Fig. 3E. It can be observed that the level of trypsin needed to digest 50% of the protein is two times higher for AmtA WT and AmtA M2 than for AmtA M1 (Fig. 3E). These data reveal that AmtA M1 is more susceptible to trypsin than AmtA WT and AmtA M2, suggesting a difference in conformation between AmtA WT and its derivatives.

The differences in the recovery rates after photobleaching and sensitivities to trypsin digestion of AmtA M1 compared to those of AmtA WT and AmtA M2 suggest that the conformation of AmtA M1 is different from those of AmtA WT and AmtA M2. On the other hand, we could not detect a discernible conformational difference between AmtA WT and AmtA M2 using the above-described approaches. That is, AmtA WT and AmtA M2 showed similar behaviors in their recovery rate and sensitivity to protease, while they clearly differed in their functionality.

The role of synonymous mutations manifests mainly at two different levels: (i) at the level of mRNA stability or structure and (ii) at the level of protein conformation. Moreover, the mRNA structure or stability can indirectly impact the protein conformation. To assess which one of the above-mentioned situations is possible, we calculated the free energy change (Δ*G*) values of the WT and mutant mRNAs using RNAstructure Fold online software (50). The Δ*G* values for *amtA WT*, *amtA M1*, and *amtA M2* are −338.1, −340, and −339.7, respectively. This change is unlikely to cause any significant alteration in the stability of the mRNA. Thus, it is unlikely that the mutations altered the mRNA structure and thereby impacted the protein conformation. The second level at which synonymous mutations alter protein functionality is by changing translational kinetics, which affects cotranslational folding, thereby causing a conformational change. Given that a nonsynonymous change is also required for functionality, we are inclined to suggest that synonymous mutations introduce a conformational change by altering translational kinetics. There is sufficient evidence in the literature to suggest that translational kinetics play a pivotal role in dictating conformation (45, 51–53).

### AmtA M1 and AmtA M2 show different sensitivities to methylamine toxicity and distinct mechanisms of ammonium transport.

**FIG 4:**
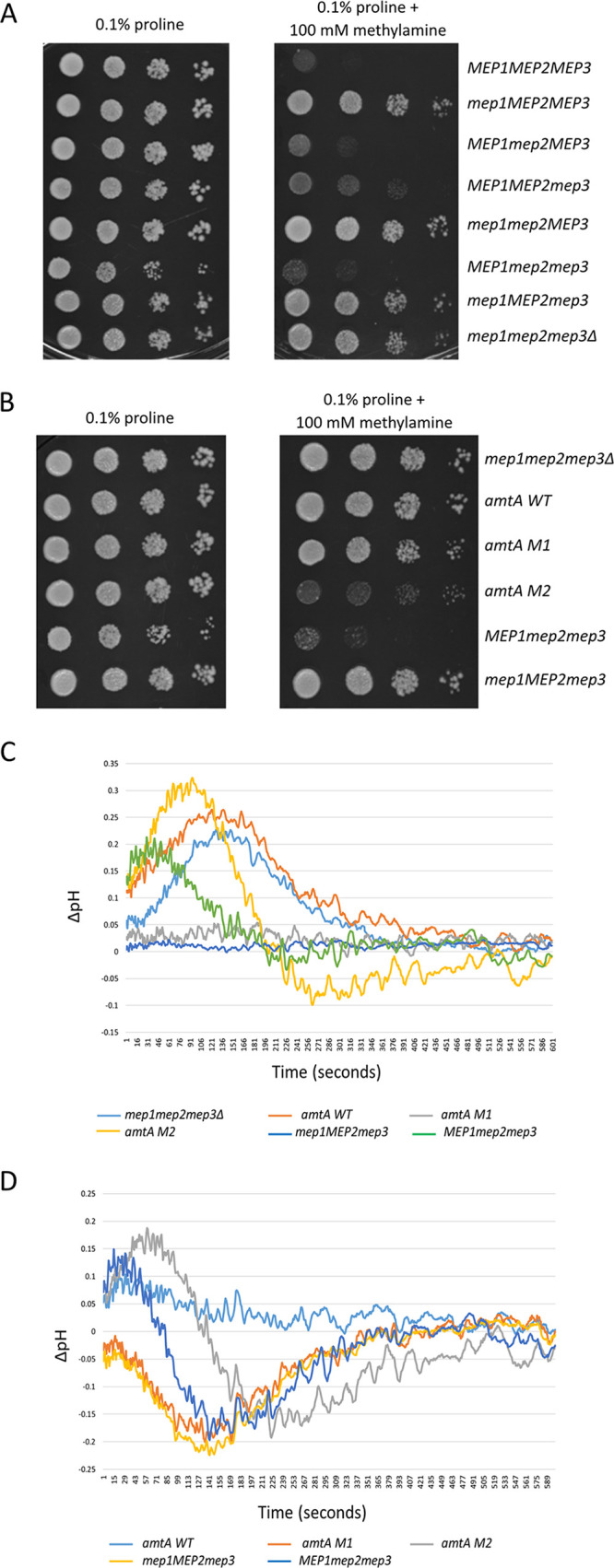
AmtA M1 and AmtA M2 differ in their ammonium transport mechanisms and methylamine sensitivities. (A) Growth of strains on methylamine. Five microliters of strains pregrown in 0.1% proline medium to a cell density of 10^7^ cells/mL was serially diluted and spotted onto 0.1% proline medium (left) and synthetic minimal medium with 0.1% proline and 100 mM methylamine (right). The growth pattern was photographed after 4 days of incubation at 30°C. (B) Growth of strains on methylamine. Five microliters of strains pregrown in 0.1% proline medium to a cell density of 10^7^ cells/mL was serially diluted and spotted onto 0.1% proline medium (left) and synthetic minimal medium with 0.1% proline and 100 mM methylamine (right). The growth pattern was photographed after 4 days of incubation at 30°C. (C) Change in the intracellular pH after ammonium addition as a function of time. The ΔpH (intracellular pH of the strain exposed to ammonium − intracellular pH of the strain exposed to water) was measured using pH-sensitive GFP as a function of time and is plotted for different strains. (D) Change in the intracellular pH after ammonium addition as a function of time. The ΔpH [(intracellular pH of the strain exposed to ammonium − intracellular pH of the strain exposed to water) − (intracellular pH of the triple-deletion strain exposed to ammonium − intracellular pH of the triple-deletion strain exposed to water)] was measured using pH-sensitive GFP as a function of time and is plotted for different strains.

AmtA M1 and AmtA M2 show ammonium uptake activity, while biochemical and microscopic examinations suggest conformational differences. How does one reconcile these observations? Is it possible that the difference in the conformations is a reflection of their functional diversification? For example, S. cerevisiae Mep proteins take up methylamine albeit to different extents (15, 54). It was also shown previously that a strain expressing only *MEP1*, but not strains expressing *MEP2* alone or *MEP3* alone, confers sensitivity to methylamine when cells are grown in 0.1% proline as the sole source of nitrogen (7, 15) (Fig. 4A). We confirmed this observation (Fig. 4A). Furthermore, the inability of Mep2 to confer methylamine toxicity is attributed to the conserved histidine (H194) that is present in Mep2 but not in Mep1, which has glutamate at the corresponding position instead of histidine (Fig. S5) (15). Accordingly, the ability of Mep1 to cause methylamine toxicity is abolished by replacing E194 with histidine. Substituting glutamate instead of histidine at position 194 in Mep2 confers methylamine toxicity. These studies point out that glutamate at position 194 corresponding to the conserved histidine is crucial for imparting methylamine toxicity (15).

AmtA WT, AmtA M1, and AmtA M2 have histidine at the conserved position 194 (Fig. S5). Based on the above-described results, AmtA WT and its mutant derivatives are not expected to confer methylamine toxicity to the triple-deletion strain. To investigate whether the above-mentioned expectation holds, we carried out a similar analysis with strains expressing *amtA WT*, *amtA M1*, and *amtA M2*. As positive and negative controls, we included strains expressing *MEP1* and *MEP2* individually (Fig. 4B). It was observed that AmtA M2 confers methylamine toxicity, while AmtA WT and AmtA M1 do not confer methylamine toxicity. These observations suggest that the glutamate at position 194 *per se* (Fig. S5) is not sufficient for conferring methylamine toxicity.

Since AmtA M1 and AmtA M2 show different responses to methylamine toxicity, we were curious to see whether AmtA M1 and AmtA M2 behave differently with respect to the mechanism of ammonium transport. We used a ratiometric GFP, which is sensitive to pH, to measure the cytosolic pH as a function of the exposure of cells to an ammonium pulse at pH 6.1, as demonstrated in a recent study (18). In that study, intracellular pH was measured as a function of ammonium transport using pHluorin as a probe in S. cerevisiae. Yeast cells grown at pH 6.1 were used to measure the effect of ammonium transporter Mep1, Mep2, and Mep2 mutants on the cytosolic pH of the cells after the addition of ammonium at a concentration of 2 mM (18). We followed a similar protocol where cells were grown in a medium of pH 6.1 containing 0.1% proline as the sole nitrogen source. At an optical density at 600 nm (OD_600_) of 1.0, ammonium was added to a concentration of 2 mM, and the intracellular ratiometric fluorescence was recorded as a function of time.

The triple-deletion strain served as a negative control. Figure 4C shows the profiles of all strains after subtracting the values obtained from the control (addition of water instead of ammonium). This reflects the change in the intracellular pH due only to the presence of ammonium in the medium. The triple-deletion strain and the *amtA WT* strain responded similarly. The patterns of pH change exhibited by strains expressing *amtA M1* and *MEP2* were similar. On the other hand, the patterns of pH change exhibited by strains expressing *amtA M2* and *MEP1* strains were similar, although they were different in magnitude. Figure 4D provides the profiles of intracellular pH after subtracting the values obtained from the triple-deletion strain. In effect, this would get rid of any change in the pH profile caused by any phenomena other than ammonium transport. It can be observed that the *amtA M1* and *MEP2* strains respond identically. This is not surprising as they both transport ammonium and are not sensitive to methylamine. On the other hand, strains expressing *amtA M2* and *MEP1* transport ammonium and are sensitive to methylamine, and their intracellular pH responses to ammonium addition are similar. Thus, we demonstrate that a functional equivalence exists between AmtA M1 and Mep2 as well as between AmtA M2 and Mep1, even with respect to intracellular pH profiles.

The mechanisms of the translocation of ammonium are measured using either a homologous system or a heterologous system. In a heterologous system, the transporters are expressed routinely in Xenopus laevis oocytes, and electrophysiological experiments are performed. A second approach has been to reconstitute purified ammonium transporters into liposomes and then monitor their translocation (18, 55–57). In either case, the current is measured at a fixed voltage as a function of ammonium translocation. It has been observed that ammonium translocation causes a change in the current in as early as a few seconds, and thereafter, it reaches a steady state. This pattern was also observed when the intracellular pH was monitored as a function of time in response to ammonium addition. Using both approaches, it was inferred that Mep1 and Mep2 translocate ammonium through electrogenic and electroneutral mechanisms, respectively (18). Based on the above-described results and the functional equivalence between AmtA M2 and Mep1 presented here, it is tempting to speculate that AmtA M1 translocates ammonium through an electroneutral mechanism, while AmtA M2 translocates ammonium through an electrogenic mechanism. However, one needs to be cautious before extrapolating these results since contradictory results have been reported for the mechanism of ammonium transport, and a consensus on this topic has not been reached (13–15, 18, 57). Nevertheless, the results reported here clearly provide additional experimental avenues to address the above-mentioned issues.

## DISCUSSION

We set out to study the structure-function relationship of the D. discoideum ammonium transporter *amtA* by employing a complementation approach in S. cerevisiae. Our attempt yielded the following unexpected results: (i) while ammonium transporters from evolutionarily distant organisms are known to complement the triple-deletion S. cerevisiae strain for growth on low-ammonium medium (14, 28–30), *amtA* failed to complement; (ii) at least three substitutions, both synonymous and nonsynonymous, are necessary to confer functionality on *amtA* when expressed in the triple-deletion strain; and (iii) *amtA M2* had acquired the abilities to confer growth on low-ammonium medium and to confer toxicity in the presence of methylamine. These results do not necessarily suggest that single or double mutations are insufficient to complement the triple-deletion strain to grow on low-ammonium medium. It is possible that the library that we generated might not represent clones with single or double substitutions at a sufficiently high frequency or such mutants with partial activity would have escaped our stringent screening protocol, or it could be a combination of the above-mentioned possibilities. It appears that our library contained a preponderance of clones with multiple substitutions, although we *a priori* did not intend to use an error-prone PCR protocol that gives a higher frequency of mutations per ORF. Contrary to common expectations, it has been demonstrated that EP-PCR-generated libraries often contain clones with higher rates of substitutions and allow the protein to traverse distances in sequence space, which provides alternate avenues for exploring new functions (58).

Observations that the single or other combinations of pairs of mutant intermediates of *amtA M1* and *amtA M2* were nonfunctional and the fact that *amtA M2* also confers methylammonium toxicity in addition to ammonium transport make it imperative to look at the possible mechanistic basis of the observations reported here. We hypothesize that the nonfunctionality of *amtA WT* in S. cerevisiae arises mainly because AmtA, being a membrane protein, is too sensitive to alterations in translational speed. A fundamental reason for this could be that translation, folding, and membrane integration are coupled processes (59) and therefore involve multiple equilibria (60). Any disturbance in the kinetics of any one of the processes is sufficient to impair the overall outcome. It is pertinent to mention that the functional ammonium transporters exist as trimers, probably making them even more susceptible to changes in translational speed. Thus, any parameter that alters translational kinetics is likely to cause a nonfunctional protein product. Considering the above-mentioned factors, it is not surprising that many of the detailed studies on the effect of synonymous substitutions on protein function have come from membrane proteins such as multidrug resistance protein (MDR) and cystic fibrosis transmembrane conductance regulator (CFTR) (51, 53, 61–63).

We hypothesize that the upper limit of the codon preference to maintain the optimal translation speed of ammonium transporters in S. cerevisiae is set by the *MEP2* codons (Fig. 1A). According to this hypothesis, a codon preference of ammonium transporters above but not below this limit will result in a nonfunctional protein product. This hypothesis is based primarily on the observation that ammonium transporters from evolutionarily distant organisms whose codon preferences are lower than that of *MEP2* (Fig. 1A) have been found to be functional (14, 28, 29). The higher codon preference of D. discoideum ammonium transporters in S. cerevisiae is not unique to *amtA* but appears to be a general feature of all ammonium transporters of D. discoideum (see Fig. S6 in the supplemental material). Furthermore, a closer look at the sequences of *amtA M1* and *amtA M2* indicates that the substitutions are not localized to any specific part of the primary sequence but are spread throughout the primary sequence (Fig. S5). This suggests that the effect of these substitutions is generic and not localized to a specific part of the sequence. The above-described hypothesis has a clear-cut prediction in that the ammonium transporters that are functional in S. cerevisiae, such as E. coli amtB, would become nonfunctional if their translational speed is increased beyond what is set by *MEP2*.

The most intriguing result of this study is the observation that both synonymous and nonsynonymous substitutions are necessary to confer ammonium transport on the independently isolated mutants *amtA M1* and *amtA M2*. The role of single nucleotide mutations leading to nonsynonymous substitutions has been the mainstay of protein evolution (64). However, it is now well documented that synonymous substitutions are not neutral and play a significant role in protein biogenesis by altering translational fidelity, the translational rate, and cotranslational folding and therefore can eventually have profound effects on organismal fitness (45, 51, 53, 65, 66). It has been reported that the mechanistic basis of an increase in fitness caused by synonymous and nonsynonymous mutations could not be discriminated (67). More recently, it has been demonstrated that even the preceding and the succeeding codon contexts have significant roles in determining the translation speed (63, 68). Much of our knowledge of the biological consequences of synonymous substitutions comes from studies where the effect of synonymous codons is determined while keeping the rest of the amino acid sequence context intact (51, 61, 62). In a real scenario, protein suffers from mutational events, after which functionally important amino acid residues are selected. While the substitutions of amino acids in orthologues can be rationalized using mutational and biophysical techniques, the underlying chemical logic for the conservation of codons in the context of various sequences is difficult to decipher (64). That is, in the absence of an *a priori* theoretical understanding, the predictive power to empirically determine the effect of synonymous/nonsynonymous substitutions on fitness under the conditions of various sequence contexts is lacking.

How do we explain the mechanistic basis of the combined role played by synonymous and nonsynonymous substitutions? Recent studies clearly demonstrated an epistatic relationship between synonymous and nonsynonymous substitutions in the CFTR protein (62, 63). However, we were unable to detect epistatic effects between various substitutions within *amtA M1* and *amtA M2* either because our system is not sensitive enough to detect minor activity that could be associated with such intermediates (single, double, or triple mutations, etc.) or there is no epistasis between the mutant pairs. During the early days of molecular evolutionary studies, it was proposed that a wide range of amino acids can be substituted without altering functionality only at some sites but not all sites (69). That is, the evolution of different sites in a protein is constrained to different extents because of the cooperative nature of interactions between any pair of amino acids, a phenomenon commonly termed intragenic epistasis, first experimentally observed in tryptophan synthase (70). Alternatively, the results presented here are an example of higher-order epistasis wherein the effect of a three-way mutant cannot be explained by the individual or pairwise effect of its constitutive mutants. In fact, more often, the interaction can be among three or more amino acids, a phenomenon first observed in lysozyme (71). Higher-order epistasis in protein evolution has been intensely investigated in the past 2 decades to understand protein evolution, with the larger goal of mapping genotype to phenotype (72–74).

Epistasis as discussed above is due mainly to nonadditivity resulting from the accumulation of destabilizing mutations (75–78). In contrast to the results described above, a recent study (79) invokes ensemble epistasis, wherein interactions between mutations differentially alter the relative concentrations of conformations of an ensemble, giving rise to nonadditive effects. A precondition for ensemble epistasis to be manifested is that the protein populates with three or more conformations. The above-mentioned study predicted that a significant fraction of mutant pairs exhibit ensemble epistasis (79). It was reported previously that higher-order substitutions increase the conformational ensemble in wild-type Gal3p, an allosteric signal transducer of the *GAL* genetic switch of S. cerevisiae (80). Thus, ensemble epistasis driven by higher-order substitutions may be at play in conferring the ammonium transport function to *amtA* mutants when expressed in S. cerevisiae.

**FIG 5:**
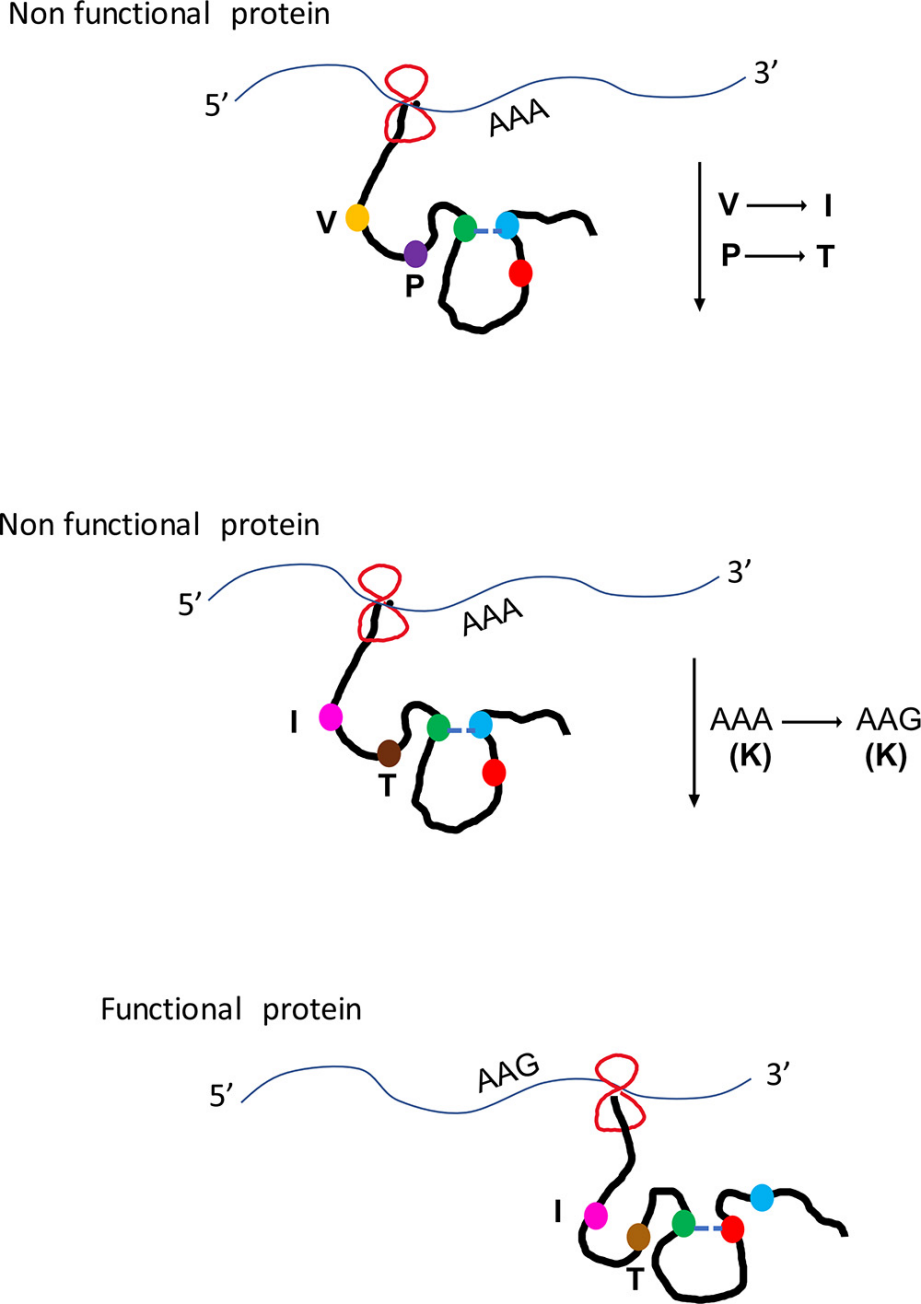
Representative mechanism of AmtA M2 gain of function in S. cerevisiae. Green, red, and blue spheres represent hypothetical amino acid residues. Yellow and purple spheres represent valine and proline, respectively.

Figure 5 depicts a general scenario of events that could occur during *amtA M2* translation. Green, red, and blue spheres represent hypothetical amino acid residues. In the functional protein, green interacts with red. Because of the increased translational speed, green interacts with blue instead of red, leading to a nonfunctional protein. This nonspecific interaction is suppressed and the specific interaction between green and red is restored because of P129T and V334I substitutions. However, P129T and V334I substitutions by themselves cannot suppress the nonspecific interaction. This has to be accompanied by a synonymous change from AAA to AAG. In S. cerevisiae, the number of tRNA genes corresponding to AAA is 14, compared to 7 for AAG (81). Possibly, this synonymous substitution, in addition to the P129T and V334I substitutions, aids in overcoming the nonspecific interaction by decreasing the speed of translation. Since a direct interaction between threonine and isoleucine is not possible, we suspect that their effect is mediated through alterations in the translation speed.

It was suggested previously that to understand the role of epistasis in protein evolution, measurements have to be made in the native host (82). However, we contend that since most of the studies on epistasis are performed in the native host or hosts that are permissive for the functional expression of proteins, the interaction between synonymous and nonsynonymous substitutions that otherwise exists would have escaped detection. For example, as discussed in the introduction, the triple-deletion strain serves as a permissive host for the functional expression of ammonium transporters. That is, studying *amtA* would not have yielded the results presented here if yeasts were to be a permissive host of *amtA*. The biological meaning of such context-dependent behaviors of synonymous mutations is highlighted in a previous study where the authors demonstrated that synonymous substitutions can function in a tissue-specific manner (53). Based on the above-described results, we suggest that complementation across incompatible genomes can be a powerful approach for deciphering the trajectory of protein evolution.

A large number of computational techniques have been developed (83, 84), often with great success (85, 86), to predict the evolutionary pattern of nonsynonymous substitutions in orthologues. An implicit assumption of these approaches is that orthologues encounter similar selective constraints. This assumption is less likely to hold for genes acquired through horizontal gene transfer (HGT). For example, a commonly encountered selective constraint during HGT is codon bias (87, 88). This selective constraint is overcome by amelioration, a process (89, 90) that involves the accumulation of synonymous substitutions in the gene under selection. Besides, there are indications that the host genome could also undergo compensatory changes such as variation in the tRNA copy number or mutations in anticodons (91, 92), thereby alleviating the fitness cost. It was recently reported that in bacterial populations, HGT can increase the potential genetic diversity without having any selective value (93). In light of the above-mentioned results, our study hints at the possibility that the fitness cost imposed by codon bias can be alleviated through cooperative interactions between synonymous and nonsynonymous substitutions, a phenomenon that has not been considered so far as a possible mechanism of protein evolution.

The current understanding of protein evolution is shaped mainly by the Darwinian concept of descent with modification (94), wherein genetic material is vertically transmitted from parent to offspring. Darwin stated, “If it could be demonstrated that any complex organ existed which could not possibly have been formed by numerous, successive, slight modifications, my theory would absolutely break down.” This statement clearly underscores the nature of modifications relevant to Darwinian evolution. Based on theoretical and experimental observations, the above-mentioned idea is now recast in molecular terms by looking at how proteins explore the neighboring sequence space in a stepwise sequential fashion to improve functions or gain new functions (95–98). How would this idea play out when evolution occurs through horizontal/lateral gene transfer (99–101)? While HGT was initially thought to play a marginal role (102), it has now been clearly demonstrated that it has as important a role in evolution as that of the vertical transmission of genetic material (103, 104). For example, a transporter gene acquisition ratchet (105) seems to have played a dominant role in fungi in diversifying their ability to adapt to various carbon and nitrogen sources (4, 105–107). Despite the above-mentioned results, studies on molecular evolution are more implicitly based on the model of vertical gene transmission than on HGT. Therefore, it is pertinent to revisit the hypothesis that HGT played a crucial role in the evolution of universality and optimality, the defining features of genetic code (108).

Overall, our study has raised many questions that have wide-ranging ramifications in understanding protein evolution. For example, can bifunctionality (ammonium and methylamine transport) be introduced by altering translation kinetics? It has been reported that ammonium transporters also transport CO_2_ (3, 9–11), but the underlying evolutionary mechanisms that led to the above-described functional diversification have not been addressed. At a more fundamental level, what our study indicates is that synonymous and nonsynonymous substitutions can interact in many ways to confer subtle variations in protein function that natural selection can operate on. Therefore, this study should not be overlooked as an isolated example and should instead be considered a stepping stone for more detailed studies of the role of cooperation between synonymous and nonsynonymous substitutions in protein evolution. Understanding the role of cooperation between synonymous and nonsynonymous substitutions in protein evolution has important implications not only for studying protein evolution but also for deciphering the role of single nucleotide polymorphisms (SNPs) in genetic diseases (109–111) to obtain functional proteins when expressed in heterologous systems (112).

## MATERIALS AND METHODS

### Medium components and growth conditions.

Yeast cultures were grown in yeast extract-peptone-dextrose (YPD) medium and the appropriate synthetic complete glucose medium. Bacterial (E. coli) cultures were grown in Luria broth (LB) agar and liquid media and complemented with ampicillin at a concentration of 75 μg/mL whenever required. Yeast extract, LB, and LB agar were purchased from Hi-Media Laboratories. Bacto peptone and yeast nitrogen base (YNB) were obtained from Difco (Detroit, MI).

YPD medium contained 0.5% yeast extract, 1% peptone, 2% dextrose, and 2% agar.

Synthetic complete and dropout glucose media contained 2% glucose, ammonium sulfate plus YNB without amino acid mix (3:1) (0.66 g/100 mL) or with amino acid mix (0.05 g/100 mL), and 2% agar.

Amino acid mix contained adenine at 0.8 g, uracil at 0.8 g, tryptophan at 0.8 g, histidine at 0.8 g, arginine at 0.8 g, methionine at 0.8 g, leucine at 1.2 g, tyrosine at 1.2 g, lysine at 1.2 g, phenylalanine at 2.0 g, threonine at 8.0 g, aspartic acid at 4.0 g, valine at 1.5 g, and isoleucine at 0.3 g. The complete amino acid mixture contains all of the above-mentioned amino acids and nitrogen bases. A particular dropout amino acid mixture lacks the indicated specific component.

Low-ammonium medium contained 500 μM ammonium sulfate, 0.17% yeast nitrogen base (without ammonium sulfate), 2% glucose, and 2% agar.

For media containing different ammonium concentrations, only the ammonium sulfate concentration varied accordingly.

Medium with 0.1% proline was composed of 0.1% proline, 0.17% yeast nitrogen base (without ammonium sulfate), 2% glucose, and 2% agar.

Medium containing 0.1% proline and 100 mM methylamine was composed of 0.1% proline, 100 mM methylamine hydrochloride, 0.17% yeast nitrogen base (without ammonium sulfate), 2% glucose, and 2% agar.

### DNA modification, electrophoresis, and Western blotting.

Tris-EDTA, amino acids, and the reagents used for agarose gel electrophoresis were obtained from Sigma-Aldrich USA. Restriction enzymes were obtained from Fermentas, and DNA polymerases were obtained from New England BioLabs (NEB); all other chemicals used were of analytical grade. Pma1 was probed with mouse Pma1 monoclonal antibody (clone 40B7), yEGFP was probed using anti-GFP antibody from mouse (catalogue number 11814460001; Roche), and anti-mouse IgG-alkaline phosphatase antibody (catalogue number A9316) was used as the secondary antibody.

### Plasmid isolation from E. coli.

The plasmid was isolated from E. coli by the alkaline lysis method as described previously (113).

### Yeast transformation.

A single colony was inoculated into 5 mL of YPD medium and incubated at 30°C with shaking until the OD_600_ reached 0.5. Cells from 1.5 mL of the culture were harvested by centrifugation at 13,000 rpm for 1 min. Cells were washed three times with 200 μL of TE (Tris-EDTA) buffer (10 mM Tris, 1 mM EDTA [pH 8.0]). Cells were washed two times with 200 μL of 100 mM lithium acetate. Cells were resuspended in 150 μL of 100 mM lithium acetate, and DNA was added to these cells along with 5 μL of single-stranded DNA. Cultures were incubated at 30°C for 5 min. One milliliter of 40% polyethylene glycol 3350 (PEG 3350) was added to this mixture, and tubes were incubated at 30°C for 1 h. After 1 h of incubation, cells were subjected to a heat shock at 42°C for 20 min. Cells were harvested by centrifugation at 13,000 rpm for 5 min and washed twice with TE buffer. Cells were then resuspended in 100 μL of TE buffer and plated onto the appropriate medium.

### Yeast genomic DNA isolation.

A single colony of yeast was inoculated into 5 mL of YPD broth and grown at 30°C for approximately 20 h. Cells from this culture were pelleted in a microcentrifuge tube. The pellet was resuspended in 200 μL of lysis buffer (2% Triton X-100, 1% SDS, 100 mM NaCl, 10 mM Tris-HCl [pH 8.0], and 1 mM EDTA [pH 8.0]). Totals of 0.3 g of acid-washed glass beads and 200 μL of phenol-chloroform-isoamyl alcohol mix were added to the above-mentioned tubes. Tubes were then vortexed for 3 to 5 min at intervals of 30 s with intermittent incubation on ice. After the addition of 200 μL of TE buffer, tubes were centrifuged for 15 min at 13,000 rpm at 4°C. The aqueous layer was transferred to a fresh tube, and DNA was precipitated by the addition of 1 mL of absolute ice-cold ethanol. Samples were allowed to precipitate for 5 min at 4°C and then centrifuged for 10 min at 13,000 rpm at 4°C. DNA pellets were air dried, resuspended in 400 μL of TE buffer with 3 μL of RNase (10 mg/mL), and kept for 5 min at 37°C. After this, DNA was reprecipitated by the addition of 10 μL of 4 M ammonium acetate and 1 mL of absolute ice-cold ethanol. After centrifugation at 4°C for 10 min at 13,000 rpm, the DNA pellet was dissolved in 25 μL of TE buffer.

### Error-prone PCR.

The error-prone PCR protocol used was performed as reported previously (80, 114, 115), with the modifications mentioned below. The PCR conditions for *amtA* amplification comprised an initial denaturation step at 95°C for 7 min followed by 30 cycles of denaturation at 95°C for 1 min, annealing at 55°C for 1 min, and extension at 72°C for 3 min. A final extension step was carried out at 72°C for 14 min. In order to introduce random mutations, dTTP and dCTP were used at a concentration of 4 mM, while dATP and dGTP were used at a concentration of 2 mM (114, 115). PCR was carried out using different concentrations of MnCl_2_ to determine the optimal concentration of MnCl_2_ required for the maximum yield of the PCR product. This was necessary to obtain the maximum number of *in vivo* recombinants in S. cerevisiae. It was observed that the yield of the PCR product was maximum at concentrations of 0.35 mM and 0.45 mM MnCl_2_ (data not shown). Two independent error-prone PCR libraries were generated using 0.35 and 0.45 mM MnCl_2_.

### Disruption/integration of ORFs.

The specific ORF was disrupted/integrated into a strain using KanMX4 cassette scoring for Geneticin resistance or using hphMX6 cassette scoring for hygromycin resistance. Primers were used to amplify the KanMX4 cassette from the pUG6 plasmid or the hphMX6 cassette from the pUG75 plasmid. Transformants were selected onto a YPD plate containing the antibiotic Geneticin or hygromycin. Putative transformants were tested for gene disruption/integration by diagnostic PCR using an internal primer of the KanMX4 cassette or the hphMX6 cassette and also using primers that give a product if the ORF is disrupted/integrated, while the wild-type strain will not give any PCR product.

### Ammonium estimation assay.

Strains were grown in medium containing 0.1% proline as the sole nitrogen source until the OD_600_ reached 1.0. Cells were collected, washed twice with sterile distilled water, and transferred to medium containing 500 μM ammonium and 0.1% proline as the nitrogen source. The external ammonium concentration was estimated as a function of time for 3 h using the below-mentioned principle (ammonia assay kit; Sigma-Aldrich). At each time point, cells were collected and filtered, and the filtrate was taken for estimating the ammonium concentration. Ammonia reacts with α-ketoglutarate and reduced NADPH in the presence of glutamate dehydrogenase (GDH) to form l-glutamate, oxidized NADP^+^, and water. The decrease in the absorbance at 340 nm due to the oxidation of NADPH is proportional to the ammonium concentration.

### Cytosolic pH measurement using pHluorin.

**(i) Calibration.** Strains were grown in medium containing 0.1% proline as the sole nitrogen source until the OD_600_ reached 1.0, collected, and resuspended in phosphate-buffered saline (PBS) buffer containing 100 μg/mL digitonin. This mixture was agitated gently for 15 min using a circular rotor. Cells were pelleted and resuspended in Na_2_HPO_4_-citrate buffer with the pH varying from 4.8 to 8.0. Fluorescence was measured at two excitation wavelengths, 395 nm and 475 nm, with the emission wavelength at 510 nm using a Jasco FP 8500 spectrofluorometer. A nonlinear curve (polynomial with order 2) was plotted for each pH against the *I*_395/475_ fluorescence value.

**(ii) Sample pH measurement.** Cells were grown in medium containing 0.1% proline as the sole nitrogen source until the OD_600_ reached 1.0. Fluorescence was measured at two excitation wavelengths, 395 nm and 475 nm, with the emission wavelength at 510 nm for 10 min (600 s) after the addition of 2 mM ammonium or water. The *I*_395/475_ fluorescence value was calculated by extrapolation from the calibration curve. The pH profile obtained for each strain when water was used as a negative control was subtracted from the values obtained for cells exposed to ammonium. The same analysis was done for the triple-deletion strain in the presence of water as well as ammonium. ΔpH represents the intracellular pH profile of the corresponding strains after subtracting the triple-deletion strain values (18).

### Fluorescence recovery after photobleaching.

Cells were grown in medium containing 0.1% proline as the sole nitrogen source for 22 h until the OD_600_ reached 3.0. One milliliter of cells was pelleted and collected. Cells were immobilized using 1% agarose pads and observed using a laser scanning microscope (LSM 780; Carl Zeiss, Germany) at a ×100 magnification. The membrane with yEGFP-tagged protein was subjected to fluorescence recovery after photobleaching (FRAP) for 60 s using an argon ion laser (488 nm). The region of interest (ROI) is the area where bleaching is done at 100% laser intensity for a fixed area at the membrane. The negative control is the membrane area where bleaching is not done. The background is the background fluorescence of the sample slide. The normalized fluorescence intensity was analyzed using the double-normalization method used in the easyFRAP-web online tool (116).

### Plasma membrane isolation.

Cells were grown in medium containing 0.1% proline as the sole nitrogen source for 22 h until the OD_600_ reached 3.0. Cells were harvested by spinning at 13,000 rpm for 15 min at 4°C. Cells were washed, resuspended in lysis buffer (50 mM Tris base [pH 7.4], 0.5 M HCl, 20% [vol/vol] glycerol, 2 mM phenylmethylsulfonyl fluoride [PMSF], 0.05 mg/mL protease inhibitor cocktail), and disrupted by beat beating using glass beads (five times for 1 min each with 1-min intervals on ice). The cell lysate was spun at 200,000 × *g* for 90 min using a 100 Ti rotor in a Beckman Coulter XPN 100 ultracentrifuge to obtain the plasma membrane fraction as the pellet. The pellet was resuspended in membrane resuspension buffer (50 mM Tris base [pH 7.4], 0.2 M HCl, 10% [vol/vol] glycerol, 2 mM PMSF, 0.05 mg/mL protease inhibitor cocktail).

### Trypsin digestion.

The concentration of protein in a sample was estimated by SDS-PAGE using Pma1 as a loading control. The specified concentration of trypsin was added to the membrane fraction containing the desired protein (membrane resuspension buffer did not have PMSF and protein inhibitor cocktail) and kept at 37°C for 5 min. One microliter of 10 mg/mL of the trypsin inhibitor was added to the sample to stop the trypsin activity.
