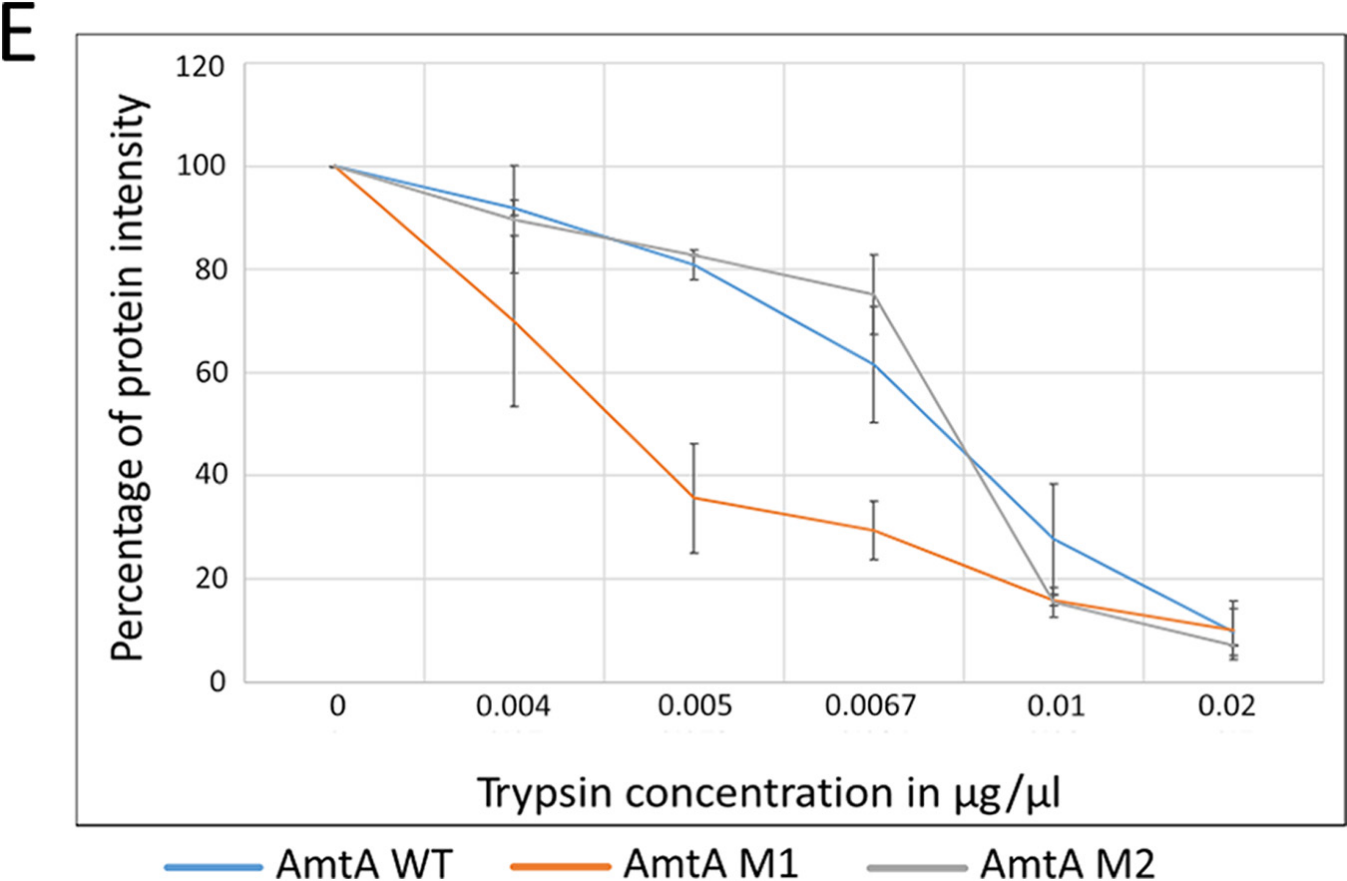# Erratum for Densi et al., “Synonymous and Nonsynonymous Substitutions in Dictyostelium discoideum Ammonium Transporter *amtA* Are Necessary for Functional Complementation in Saccharomyces cerevisiae”

**DOI:** 10.1128/spectrum.01824-23

**Published:** 2023-05-18

**Authors:** Asha Densi, Revathi S. Iyer, Paike Jayadeva Bhat

## ERRATUM

Volume 11, no. 2, e03847-22, 2023, https://doi.org/10.1128/Spectrum.03847-22.

Fig. 3, panel E: The *x* axis is incorrectly labeled. The correctly labeled panel should appear as shown in this erratum.[Fig fig3]

The second sentence pertaining to panel D (“Membrane fractions of the *amtA WT*, *amtA M1*, *amtA M2*, and *MEP2* strains were subjected to trypsin digestion”) should read “Membrane fractions of the *amtA WT*, *amtA M1*, and *amtA M2* strains were subjected to trypsin digestion,” and the second sentence pertaining to panel E (“Membrane fractions of the *amtA WT*, *amtA M1*, *amtA M2*, and *MEP2* strains were subjected to trypsin digestion”) should read “Membrane fractions of the *amtA WT*, *amtA M1*, and *amtA M2* strains were subjected to trypsin digestion.”

**Figure fig3:**