# Supplementary material for: Synonymous and Nonsynonymous Substitutions in Dictyostelium discoideum Ammonium Transporter amtA Are Necessary for Functional Complementation in Saccharomyces cerevisiae
Source: Microbiol Spectr. 2023 Feb 22;11(2):e03847-22. doi: 10.1128/spectrum.03847-22 (PMC10100761; doi:10.1128/spectrum.03847-22)
Supplement: Supplemental file 1 — Supplemental material. Download spectrum.03847-22-s0001.pdf, PDF file, 1.1 MB [file spectrum.03847-22-s0001.pdf]

Supplementary Figures.

Figure S1.

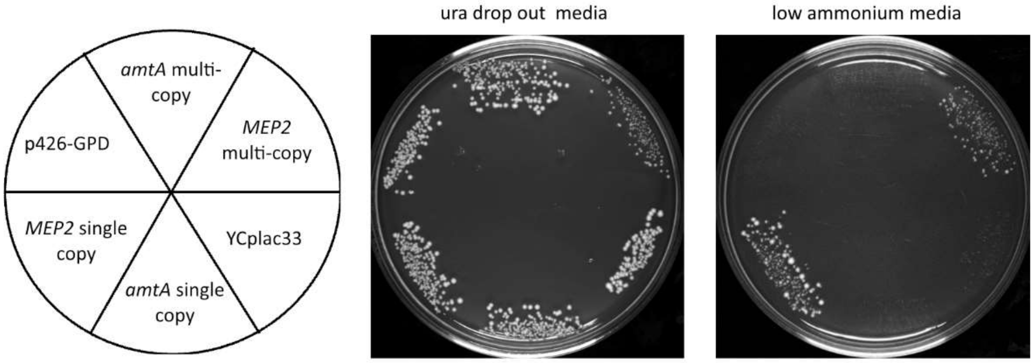

Figure S1: Growth pattern of *amtA* and *MEP2* on low ammonium at a single colony level.

5 μl of transformants of the triple deletion strain pre-grown in ura drop out glucose media containing 20 mM ammonium to a cell density of 10^7/ml were streaked onto ura drop out glucose media with 20 mM ammonium sulfate (middle panel) and low ammonium media (right panel). The growth pattern was photographed after 4 days of incubation at 30ºC. The left panel is a schematic representation of transformants bearing corresponding plasmids.

Figure S2.

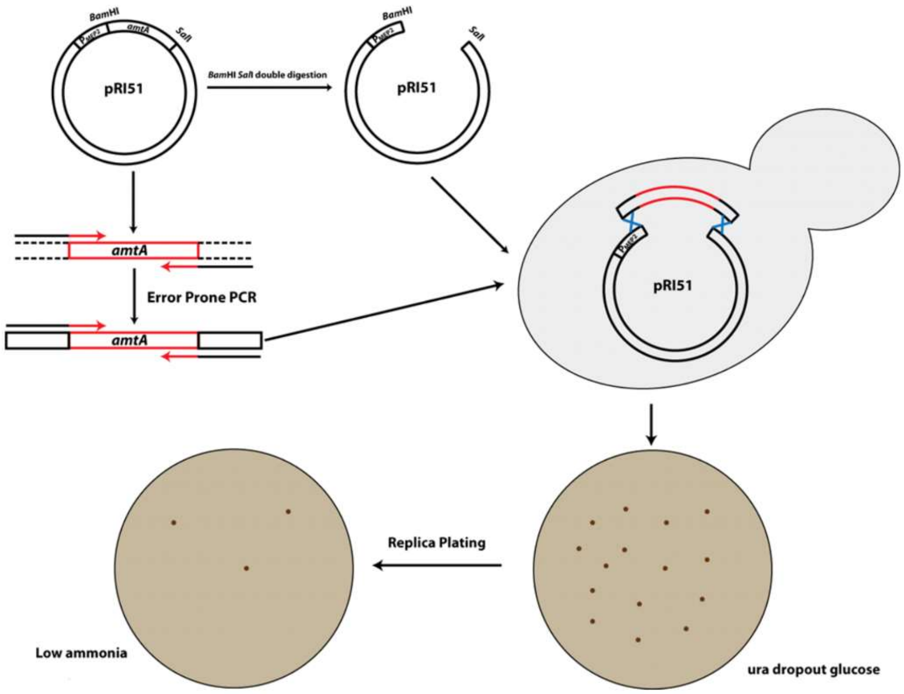

Figure S2: Schematic representation of Error prone PCR and in vivo cloning of *amtA* mutant library.

Figure S3.

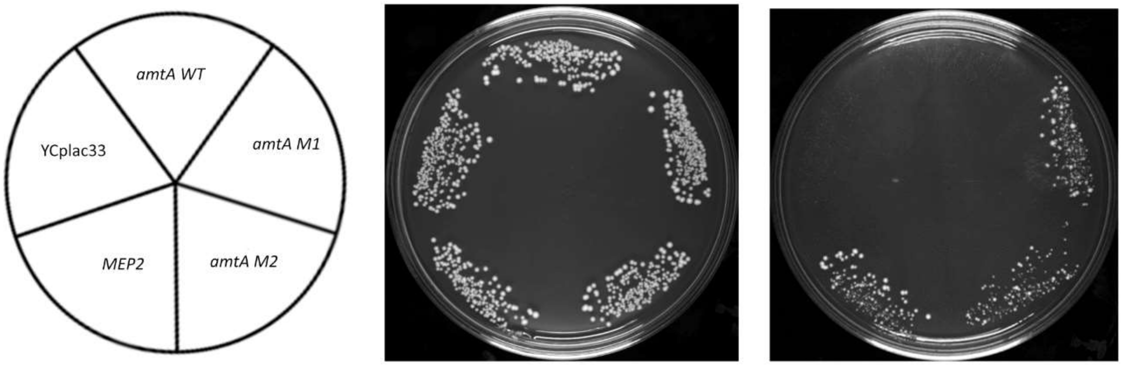

Figure S3: Growth pattern of *amtA* mutants on low ammonium at a single colony level.

5 µl of transformants of the triple deletion strain pre-grown in ura drop out glucose media containing 20 mM ammonium to a cell density of 10^7/ml were streaked onto ura drop out glucose media with 20 mM ammonium sulfate (middle panel) and low ammonium media (right panel). The growth pattern was photographed after 4 days of incubation at 30ºC. The left panel is a schematic representation of transformants bearing corresponding plasmids.

Figure S4.

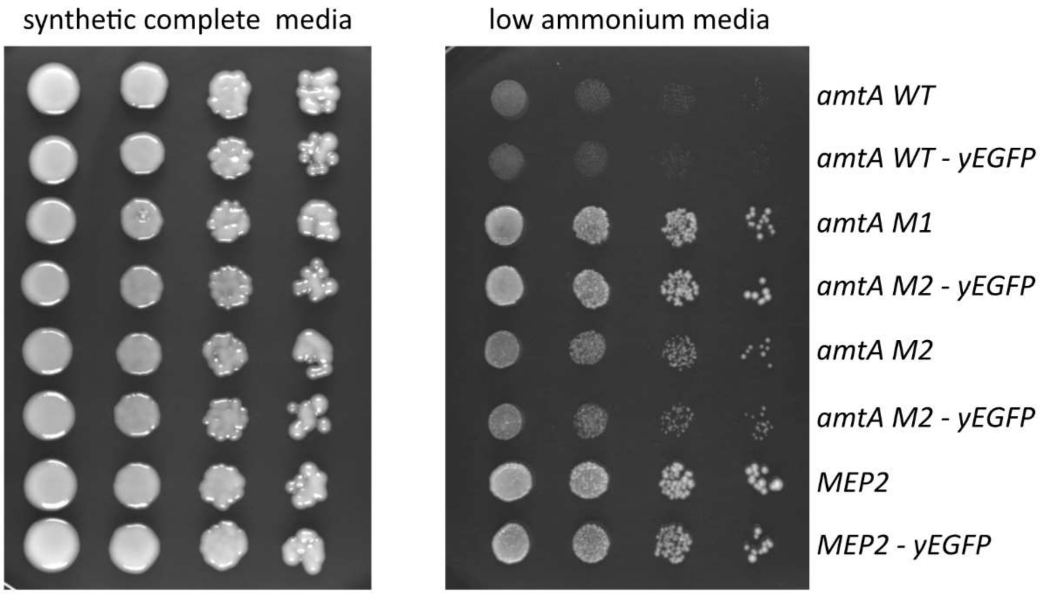

Figure S4: Growth pattern of *yEGFP* tagged strains.

5 µl of strains pre-grown in synthetic complete glucose media containing 20 mM ammonium to a cell density of 10^7/ml were serially diluted and were spotted onto synthetic complete glucose media with 20 mM ammonium sulfate (left panel) and low ammonium media (right panel). The growth pattern was photographed after 4 days of incubation at 30ºC.

Figure S5.

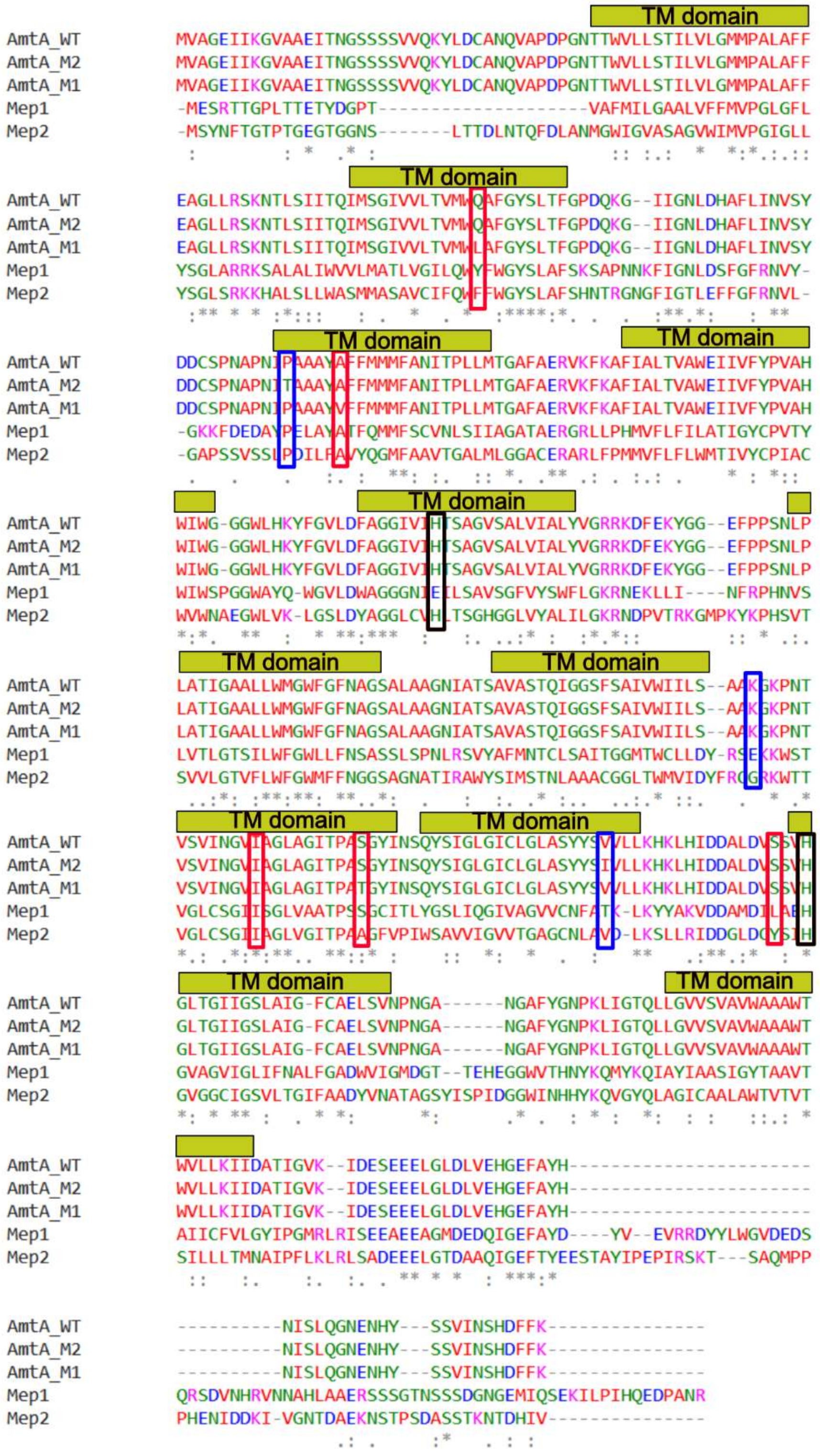
TM domain
AmtA_WT MVAGEIIKGVAAEITNGSSSSVVQKYLDCANQVAPDPGNTTWVLLSTILVLGMMPALAFF
AmtA_M2 MVAGEIIKGVAAEITNGSSSSVVQKYLDCANQVAPDPGNTTWVLLSTILVLGMMPALAFF
AmtA_M1 MVAGEIIKGVAAEITNGSSSSVVQKYLDCANQVAPDPGNTTWVLLSTILVLGMMPALAFF
Mep1 -MESRTTGPLTTETYDGPT--------------------VAFMILGAALVFFMVPGLGFL
Mep2 -MSYNFTGTPTGEGTGGNS-------LTTDLNTQFDLANMGWIGVASAGVWIMVPGIGLL
TM domain
AmtA_WT EAGLLRSKNTLSIITQIMSGIVVLTVMWQAFGYSLTFGPDQKG--IIGNLDHAFLINVSY
AmtA_M2 EAGLLRSKNTLSIITQIMSGIVVLTVMWQAFGYSLTFGPDQKG--IIGNLDHAFLINVSY
AmtA_M1 EAGLLRSKNTLSIITQIMSGIVVLTVMWLAFGYSLTFGPDQKG--IIGNLDHAFLINVSY
Mep1 YSGLARRKSALALIWVVLMATLVGILQWYFWGYSLAFSKSAPNNKFIGNLDSFGFRNVY-
Mep2 YSGLSRKKHALSLLWASMMASAVCIFQWFFWGYSLAFSHNTRGNGFIGTLEFFGFRNVL-
TM domain
TM domain
AmtA_WT DDCSPNAPNIPAAAYAFFMMMFANITPLLMTGAFAERVKFKAFIALTVAWEIIVFYPVAH
AmtA_M2 DDCSPNAPNITAAAYAFFMMMFANITPLLMTGAFAERVKFKAFIALTVAWEIIVFYPVAH
AmtA_M1 DDCSPNAPNIPAAAYVFFMMMFANITPLLMTGAFAERVKFKAFIALTVAWEIIVFYPVAH
Mep1 -GKKFDEDAYPELAYATFQMMFSCVNLSIIAGATAERGRLLPHMVFLFILATIGYCPVTY
Mep2 -GAPSSVSSLPDILFAVYQGMFAAVTGALMLGGACERARLFPMMVFLFLWMTIVYCPIAC
TM domain
AmtA_WT WIWG-GGWLHKYFGVLDFAGGIVIHTSAGVSALVIALYVGRRKDFEKYGG--EFPPSNLP
AmtA_M2 WIWG-GGWLHKYFGVLDFAGGIVIHTSAGVSALVIALYVGRRKDFEKYGG--EFPPSNLP
AmtA_M1 WIWG-GGWLHKYFGVLDFAGGIVIHTSAGVSALVIALYVGRRKDFEKYGG--EFPPSNLP
Mep1 WIWSPGGWAYQ-WGVLDWAGGGNIEILSAVSGFVYSWFLGKRNEKLLI----NFRPHNVS
Mep2 WVWNAEGWLVK-LGSLDYAGGLCVHLTSGHGGLVYALILGKRNDPVTRKGMPKYKPHSVT
TM domain
TM domain
AmtA_WT LATIGAALLWMGWFGFNAGSALAAGNIATSAVASTQIGGSFSAIVWIILS--AAKGKPNT
AmtA_M2 LATIGAALLWMGWFGFNAGSALAAGNIATSAVASTQIGGSFSAIVWIILS--AAKGKPNT
AmtA_M1 LATIGAALLWMGWFGFNAGSALAAGNIATSAVASTQIGGSFSAIVWIILS--AAKGKPNT
Mep1 LVTLGTSILWFGWLLFNSASSLSPNLRSVYAFMNTCLSAITGGMTWCLLDY-RSEKKWST
Mep2 SVVLGTVFLWFGWMFFNGGSAGNATIRAWYSIMSTNLAAACGGLTWMVIDYFRQGRKWTT
TM domain
TM domain
AmtA_WT VSVINGVIAGLAGITPASGYINSQYSIGLGICLGLASYYSVVLLKHKLHIDDALDVSSVH
AmtA_M2 VSVINGVIAGLAGITPASGYINSQYSIGLGICLGLASYYSIVLLKHKLHIDDALDVSSVH
AmtA_M1 VSVINGVIAGLAGITPATGYINSQYSIGLGICLGLASYYSVVLLKHKLHIDDALDVSSVH
Mep1 VGLCSGIISGLVAATPSSGCITLYGSLIQGIVAGVVCNFATK-LKYYAKVDDAMDILAEH
Mep2 VGLCSGIIAGLVGITPAAGFVPIWSAVVIGVVTGAGCNLAVD-LKSLLRIDDGLDCYSIH
TM domain
TM domain
AmtA_WT GLTGIIGSLAIG-FCAELSVNPNGA------NGAFYGNPKLIGTQLLGVVSVAVWAAAWT
AmtA_M2 GLTGIIGSLAIG-FCAELSVNPNGA------NGAFYGNPKLIGTQLLGVVSVAVWAAAWT
AmtA_M1 GLTGIIGSLAIG-FCAELSVNPNGA------NGAFYGNPKLIGTQLLGVVSVAVWAAAWT
Mep1 GVAGVIGLIFNALFGADWVIGMDGT--TEHEGGWVTHNYKQMYKQIAYIAASIGYTAAVT
Mep2 GVGGCIGSVLTGIFAADYVNATAGSYISPIDGGWINHHYKQVGYQLAGICAALAWTVTVT
AmtA_WT WVLLKIIDATIGVK--IDESEEELGLDLVEHGEFAYH-----------------------
AmtA_M2 WVLLKIIDATIGVK--IDESEEELGLDLVEHGEFAYH-----------------------
AmtA_M1 WVLLKIIDATIGVK--IDESEEELGLDLVEHGEFAYH-----------------------
Mep1 AIICFVLGYIPGMRLRISEEAEEAGMDEDQIGEFAYD----YV--EVRRDYYLWGVDEDS
Mep2 SILLLTMNAIPFLKLRLSADEEELGTDAAQIGEFTYEESTAYIPEPIRSKT---SAQMPP
AmtA_WT ----------NISLQGNENHY---SSVINSHDFFK---------------
AmtA_M2 ----------NISLQGNENHY---SSVINSHDFFK---------------
AmtA_M1 ----------NISLQGNENHY---SSVINSHDFFK---------------
Mep1 QRSDVNHRVNNAHLAAERSSSGTNSSSDGNGEMIQSEKILPIHQEDPANR
Mep2 PHENIDDKI-VGNTDAEKNSTPSDASSTKNTDHIV---------------

Figure S5: Multiple sequence alignment of AmtA WT, AmtA M1, AmtA M2, Mep1, and Mep2

Multiple sequence alignment of AmtA WT, AmtA M1, AmtA M2, Mep1, and Mep2 was obtained using MUSCLE, Multiple Sequence Alignment online software. His194 and His348 of Mep2 are highlighted using black boxes. The mutations in AmtA M1 and AmtA M2 are marked by red and yellow boxes respectively. The predicted transmembrane domains of AmtA WT are highlighted using green boxes labelled 'TM domain' above the sequence.

Figure S6

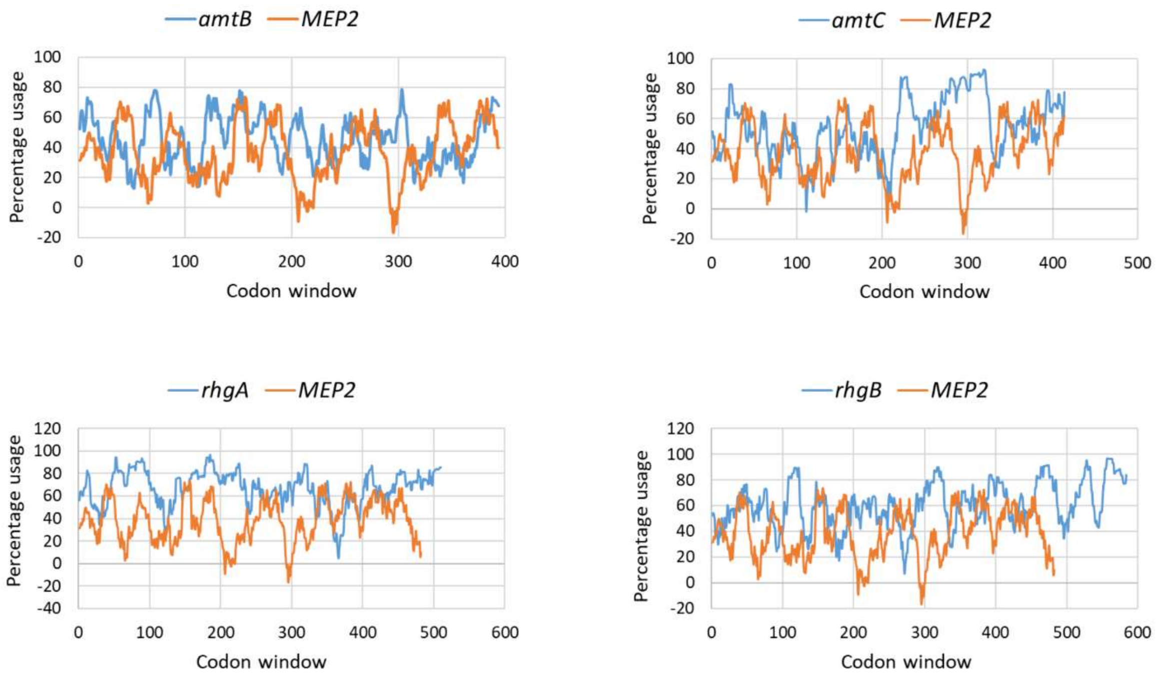

Figure S6: %MinMax of ammonium transporters expressed in *S. cerevisiae*.

%MinMax values of ammonium transporters when expressed in *S. cerevisiae* was obtained using %MinMax calculator online software [32][33]. Percentage usage of rare codons was plotted against a sliding codon window of 18 codons.
